# Risk of COVID-19-related death among patients with chronic obstructive pulmonary disease or asthma prescribed inhaled corticosteroids: an observational cohort study using the OpenSAFELY platform

**DOI:** 10.1016/S2213-2600(20)30415-X

**Published:** 2020-11

**Authors:** Anna Schultze, Alex J Walker, Brian MacKenna, Caroline E Morton, Krishnan Bhaskaran, Jeremy P Brown, Christopher T Rentsch, Elizabeth Williamson, Henry Drysdale, Richard Croker, Seb Bacon, William Hulme, Chris Bates, Helen J Curtis, Amir Mehrkar, David Evans, Peter Inglesby, Jonathan Cockburn, Helen I McDonald, Laurie Tomlinson, Rohini Mathur, Kevin Wing, Angel Y S Wong, Harriet Forbes, John Parry, Frank Hester, Sam Harper, Stephen J W Evans, Jennifer Quint, Liam Smeeth, Ian J Douglas, Ben Goldacre

**Affiliations:** a London School of Hygiene & Tropical Medicine, London, UK; b The DataLab, Nuffield Department of Primary Care Health Sciences, University of Oxford, Oxford, UK; c The Phoenix Partnership (TPP), TPP House, Leeds, UK; d NIHR Health Protection Research Unit in Immunisation, London, UK; e National Heart and Lung Institute, Imperial College London, London, UK

## Abstract

**Background:**

Early descriptions of patients admitted to hospital during the COVID-19 pandemic showed a lower prevalence of asthma and chronic obstructive pulmonary disease (COPD) than would be expected for an acute respiratory disease like COVID-19, leading to speculation that inhaled corticosteroids (ICSs) might protect against infection with severe acute respiratory syndrome coronavirus 2 or the development of serious sequelae. We assessed the association between ICS and COVID-19-related death among people with COPD or asthma using linked electronic health records (EHRs) in England, UK.

**Methods:**

In this observational study, we analysed patient-level data for people with COPD or asthma from primary care EHRs linked with death data from the Office of National Statistics using the OpenSAFELY platform. The index date (start of follow-up) for both cohorts was March 1, 2020; follow-up lasted until May 6, 2020. For the COPD cohort, individuals were eligible if they were aged 35 years or older, had COPD, were a current or former smoker, and were prescribed an ICS or long-acting β agonist plus long-acting muscarinic antagonist (LABA–LAMA) as combination therapy within the 4 months before the index date. For the asthma cohort, individuals were eligible if they were aged 18 years or older, had been diagnosed with asthma within 3 years of the index date, and were prescribed an ICS or short-acting β agonist (SABA) only within the 4 months before the index date. We compared the outcome of COVID-19-related death between people prescribed an ICS and those prescribed alternative respiratory medications: ICSs versus LABA–LAMA for the COPD cohort, and low-dose or medium-dose and high-dose ICSs versus SABAs only in the asthma cohort. We used Cox regression models to estimate hazard ratios (HRs) and 95% CIs for the association between exposure categories and the outcome in each population, adjusted for age, sex, and all other prespecified covariates. We calculated e-values to quantify the effect of unmeasured confounding on our results.

**Findings:**

We identified 148 557 people with COPD and 818 490 people with asthma who were given relevant respiratory medications in the 4 months before the index date. People with COPD who were prescribed ICSs were at increased risk of COVID-19-related death compared with those prescribed LABA–LAMA combinations (adjusted HR 1·39 [95% CI 1·10–1·76]). Compared with those prescribed SABAs only, people with asthma who were prescribed high-dose ICS were at an increased risk of death (1·55 [1·10–2·18]), whereas those given a low or medium dose were not (1·14 [0·85–1·54]). Sensitivity analyses showed that the apparent harmful association we observed could be explained by relatively small health differences between people prescribed ICS and those not prescribed ICS that were not recorded in the database (e value lower 95% CI 1·43).

**Interpretation:**

Our results do not support a major role for regular ICS use in protecting against COVID-19-related death among people with asthma or COPD. Observed increased risks of COVID-19-related death can be plausibly explained by unmeasured confounding due to disease severity.

**Funding:**

UK Medical Research Council.

## Introduction

The ongoing pandemic due to severe acute respiratory syndrome coronavirus 2 (SARS-CoV-2) has affected over 31 million people worldwide with at least 900 000 deaths due to COVID-19 as of Sept 23, 2020. People with more severe COVID-19 outcomes, including admission to hospital or death, are usually older and have pre-existing comorbidities.^1^, ^2^, ^3^, ^4^, ^5^, ^6^, ^7^ Severe outcomes are often a result of lung complications, such as acute respiratory distress syndrome and respiratory failure. However, early reports of patients with COVID-19 described an unexpectedly low prevalence of chronic respiratory conditions among those who had been admitted to hospital.^8^ Although other studies suggest that chronic lung diseases, including chronic obstructive pulmonary disease (COPD), increase the risk of severe outcomes,^5^, ^6^, ^7^ reported effect sizes for asthma have been relatively small.^5^, ^6^ These findings have led to speculation that treatments for respiratory disease, specifically inhaled corticosteroids (ICSs), might have a protective effect against SARS-CoV-2.^8^, ^9^, ^10^, ^11^

**Research in context**

**Evidence before this study**

In March, 2020, at the start of the global COVID-19 pandemic, inhaled corticosteroids (ICSs) were hypothesised to offer some protection against either infection with severe acute respiratory syndrome coronavirus 2 (SARS-CoV-2) or against severe outcomes from COVID-19, such as acute respiratory distress syndrome and respiratory failure, despite these medications being known to increase the risk of pneumonia and other respiratory tract infections. The hypothesis was based at least in part on epidemiological data showing a low prevalence of chronic respiratory disease among Chinese patients with COVID-19, although some support of a potential protective effect also came from in-vitro studies. More recently, ICS exposure was found to correlate with a lower expression of ACE2 and TMPRSS2, the entry receptors used by SARS-CoV-2, in sputum cells. A systematic review assessing whether ICSs were associated with clinical outcomes in COVID-19, severe acute respiratory syndrome, or Middle East respiratory syndrome identified no relevant studies.

**Added value of this study**

Our study was specifically designed to assess the role of routine ICS use in COVID-19-related mortality. We included two cohorts of participants: people with asthma, and people with chronic obstructive pulmonary disease (COPD), both of whom have a possible indication for ICS. Neither analysis was strongly suggestive that regular ICS therapy for asthma or COPD has a clinically important causal effect on COVID-19 mortality in either direction. Our study includes data for almost 1 million patients, making it the largest contemporary study of ICS use in COVID-19 to date. We used active comparators and multiple sensitivity analyses to quantify the effect of possible unmeasured confounding. We used open methods throughout the study with code and codelists available for examination and reuse.

**Implications of all the available evidence**

Evidence suggests there is neither a demonstrable benefit nor clear harm from ICS use against COVID-19-related mortality among people with COPD and asthma, and so no evidence supports that patients should alter their ICS therapies during the ongoing pandemic. Future observational research is likely to be subject to similar issues around unmeasured confounding, and evidence from ongoing randomised trials will provide answers regarding the role of ICS in the treatment of COVID-19 among people without asthma or COPD.

ICSs are used to reduce airway inflammation, oedema, and mucus secretions.^12^ In-vitro evidence indicates that the ICS ciclesonide can suppress SARS-CoV-2 replication,^13^ and budesonide combined with glycopyrronium and formoterol has been shown to inhibit production of cytokines in cells exposed to human coronavirus 229E.^14^ The orally or intravenously administered steroid dexamethasone has been shown to reduce the risk of death in people with severe COVID-19.^15^ Conversely, although ICSs have low systemic absorption, in people with COPD they have been associated with an increased risk of developing pneumonia^16^, ^17^, ^18^ and other systemic steroid-related adverse effects.^12^ ICS use has also been shown to impair type 1 interferon production, potentially increasing the risk of viral infections.^19^, ^20^ A systematic review of the role of ICS use in SARS-CoV-2, severe acute respiratory syndrome coronavirus 1, and Middle East respiratory syndrome coronavirus identified no relevant articles addressing this question, and whether ICS use could influence either the risk of becoming infected with SARS-CoV-2 or the clinical prognosis of COVID-19 is unclear.^9^ Two ongoing randomised controlled trials are investigating whether the clinical course of COVID-19 is affected by ICS use (NCT04331054, NCT04330586); however, these trials will not address the role of regular ICS use on the risk of SARS-CoV-2 infection and outcomes among people who have an indication for ICS use, and, specifically, whether such use might have been responsible for the under-representation of people with chronic respiratory disease in early epidemiological descriptions of COVID-19. To answer this question, we aimed to explore the association between current ICS use and COVID-19-related death among people with COPD and asthma using the OpenSAFELY platform, which runs across linked primary care electronic health record (EHR) data for approximately 40% of the population in England, UK.

## Methods

### Study design, population, and data sources

In this observational study, we extracted data for two cohorts of patients, a cohort with COPD and a cohort with asthma, from primary care EHR data linked with death data from the Office for National Statistics. The index date (start of follow-up) for both cohorts was March 1, 2020; follow-up lasted until May 6, 2020.

Primary care records managed by the EHR vendor The Phoenix Partnership (TPP; Leeds, UK) were linked to death data from the UK Office for National Statistics through OpenSAFELY, a data analytics platform created by our team on behalf of National Health Service (NHS) England^21^ to address urgent COVID-19 research questions.^6^ OpenSAFELY provides a secure software interface allowing the analysis of pseudonymised primary care patient records from England in near real-time within the EHR vendor's highly secure data centre, avoiding the need for large volumes of potentially disclosive pseudonymised patient data to be transferred offsite. This step, in addition to other technical and organisational controls, minimises any risk of re-identification. Similarly pseudonymised datasets from other data providers are securely provided to the EHR vendor and linked using a salted hash (a securely pseudonomised identification key) generated from NHS numbers. Only records with matching NHS numbers are imported, and matching quality depends entirely on the accuracy of the NHS number. We are not able to determine the quality of the linkage because we do not have access to direct identifiers from external data sources. The dataset analysed within OpenSAFELY is based on 24 million people currently registered with primary care centres using TPP SystmOne software. The data managed by TPP includes pseudonymised data such as coded diagnoses, medications, and physiological parameters. No free-text data are included.

Individuals were eligible for the COPD cohort if they were aged 35 years and older**,** had COPD, and had current or former smoking recorded any time before the index date.^22^ For primary analyses, we also required individuals to be prescribed relevant respiratory medications, detailed in the Exposures section. We excluded individuals with a previous diagnosis of any other chronic respiratory conditions, if they had asthma in the 3 years before the index date,^23^ if they had received nebulised medications in the 12 months before the index date, or if they have received a leukotriene receptor antagonist (indicating potential asthma) in the 4 months before the index date. Nebulisers are a marker of severe respiratory disease or other underlying disability that prevents the use of an inhaler. Clinically, inclusion of such patients could further emphasise differences between groups and introduce additional confounding without contributing to the study question. However, few individuals were excluded due to this criterion (appendix pp 3–4).

Individuals were eligible for the asthma cohort if they were aged 18 years or older with asthma recorded within 3 years before the index date. We excluded those with COPD or other chronic respiratory conditions before the index date, and those who were receiving a long-acting muscarinic antagonist (LAMA) without an ICS because this is an indication of possible COPD.^24^ For primary analyses, we also required individuals to be prescribed relevant respiratory medications, detailed in the Exposures section. As in the COPD cohort, those receiving nebulised medications in the 12 months before the index date were excluded. For both cohorts, people with missing data for sex or index of multiple deprivation, or with less than 1 year of primary care records were excluded (appendix p 2).

This study was approved by the Health Research Authority (Research Ethics Committee reference 20/LO/0651) and by the London School of Hygiene & Tropical Medicine ethics board (reference number 21863). No further ethical or research governance approval was required by the University of Oxford (Oxford, UK) but copies of the approval documents were reviewed and held on record. Participant consent was not required because of regulation 3(4) of the Health Service (Control of Patient Information) Regulations 2002; the legal gateways involved for General Data Protection Regulations did not require consent. All iterations of the prespecified study protocol are archived with version control and available online. Patients were not formally involved in developing the study design, which was developed rapidly in the context of a global health emergency; however, the first draft of the manuscript was sent to the Asthma UK and British Lung Foundation Partnership who provided review, comment, and suggestions from an expert patient perspective. We have also developed the OpenSAFELY website through which we invite any patient or member of the public to contact us regarding this study or the broader OpenSAFELY project.

### Exposures

In the COPD population, people issued at least one ICS prescription within 4 months before the index date either in combination with long-acting β agonist (LABA) or LABA–LAMA, or as single therapy (provided they also had at least one prescription record of a LABA in the past 4 months), were compared with those with a prescription for a LABA–LAMA (combined or as separate single therapy prescriptions) only.^24^ We did not include patients who had been given LAMA monotherapy in our primary analyses because we were expecting improved clinical comparability between the LABA–LAMA and ICS-based therapy groups, following existing National Institute for Health and Care Excellence guidance.^24^, ^25^, ^26^, ^27^ The inclusion of patients on LAMA monotherapy was assessed in a sensitivity analysis.

In the asthma population, people prescribed high-dose ICS and low-dose or medium-dose ICS during the 4 months before the index date were compared with those prescribed short-acting β agonists (SABAs) only. Exposure for people prescribed both high-dose and low-dose or medium-dose ICS was assigned according to their most recent prescription. Prescriptions for inhalers were assigned to low or medium dose or high dose using the OpenPrescribing prescribing explorer based on British Thoracic Society and Scottish Intercollegiate Guidelines Network guidance.^28^ Studies have found that a substantial proportion of people with asthma receiving SABAs only are eligible for ICS treatment,^29^ suggesting some similarity in terms of disease severity with those prescribed ICSs, and therefore we hypothesised that they could represent a reasonable active comparator group. The characteristics of all other individuals are described in the appendix (pp 5–12); however, they are excluded from regression models to avoid comparisons to individuals not prescribed drugs of interest.^30^

### Outcomes

The outcome of interest was COVID-19-related death as registered in data from the Office for National Statistics using International Classification of Diseases 10th edition (ICD-10) codes U07.1 (“COVID-19, virus identified”) and U07.2 (“COVID-19, virus not identified”) listed either as the underlying or any contributing cause of death. The U07.2 ICD-10 code is used when laboratory testing is inconclusive or unavailable but a clinical diagnosis indicates COVID-19.^31^

### Covariates

Potential determinants of exposures and outcomes were identified by reviewing the literature and through discussions with practising clinicians. Because this is a study of current users of prespecified treatments, determinants of exposures include both factors that might affect the initial choice of treatment and those that affect whether a patient remains on a specific treatment. The final list of potential confounders is in the panel. Our methods for creating codelists have been previously described;^6^ these methods included clinical and epidemiological review and sign-off by at least two authors. Detailed information on every codelist is available online.

### Statistical methods

All eligible individuals were included; we did not do sample size calculations because the sample size was fixed by the size of the database.

We summarised individuals' characteristics using descriptive statistics, stratified by exposure status. We used Kaplan-Meier plots to show time to the primary outcome, with time in study as the timescale. We dealt with the competing risk of death from non-COVID-19-realated causes by analysing the cause-specific hazard, with people dying from other causes censored at their date of death.^36^ We used Cox regression models to estimate hazard ratios (HRs) and 95% CIs for the association between exposure categories and the outcome in each population. We fitted univariable models, models adjusted for age (using restricted cubic splines) and sex, and fully adjusted models including all covariates (panel). We included the Sustainability and Transformation Partnership (an NHS administrative region) of the patient's primary care clinic as a stratification variable in fully adjusted models. We assessed a prespecified interaction between ICS exposure and age to see if we could distinguish a differential effect in groups known to be at increased risk of COVID-19-related death. We also did post-hoc analyses adjusting for each one of the prespecified comorbidities at a time (panel).

Panel **Prespecified hypothetical confounders**

All covariates were defined using primary care data only, using either information recorded in the electronic health record or diagnostic codes, or both. The analytical programs titled “study_definition”, available on our Github repository, provide the complete details of variable definitions. We used the same definitions irrespective of the exposure category an individual belonged to:

• Age (in years) as of the index date, derived from patient's date of birth
• Sex (male, female)
• BMI most recent measurement in the past decade^32^
• Indices of multiple deprivation: quintiles from index of multiple deprivation 2019^33^
• Diagnosed hypertension
• Heart disease: categorised as heart failure and other heart disease
• Diabetes: categorised as controlled (HbA_1c_ <7·5% [<58 mmol/mol]), uncontrolled (HbA_1c_ ≥7·5% [≥58 mmol/mol]), or HbA_1c_ not measured within 12 months of index date
• Cancer
• Immunosuppressive conditions: organ transplant, sickle cell anaemia, and splenectomy
• Chronic kidney disease: based on creatinine measurements within 12 months of the index date or ever having a read code for renal dialysis
• Influenza vaccination status: recorded within 6 months of the index date
• Pneumococcal vaccination status: recorded in the 5 years before the index date
• Statin use: recorded within 4 months before the index date
• Exacerbation history: different methods used for asthma^34^ and COPD population;^35^ briefly, asthma exacerbations were defined using prescriptions of oral steroids without a concurrent diagnosis indicating these were prescribed for a different indication; and COPD exacerbations were defined using an approach proposed in a previous validation study,^35^ defined as a diagnostic code for a lower respiratory tract infection or COPD exacerbation, excluding those occurring on the same date as an annual review or use of a prescription
• COPD models were additionally adjusted for a history of asthma

BMI=body-mass index. COPD=chronic obstructive pulmonary disease. HbA_1c_=glycated haemoglobin.

We assessed the proportional hazards assumption by testing for a zero slope in the scaled Schoenfeld residuals and through graphical inspection of plots of the Schoenfeld residuals against time.

To derive marginal effect estimates, we generated cumulative mortality curves standardised to adjust for differences in the covariate distributions between people exposed and not exposed to ICS. First, we fitted a flexible parametric Royston-Parmar model with the same covariates as the main Cox regression, with the baseline hazard modelled using a three (COPD) or two (asthma) degrees-of-freedom spline. We predicted the survival function from this model for every individual with the exposure set to each of the exposure levels in the COPD and asthma population in turn; we then averaged the predictions to produce population-level curves. We also used these models to estimate an adjusted absolute survival difference at the end of follow-up.

We did several sensitivity analyses. First, we split the exposure categories in the COPD population to examine the effect of ICS with LABA–LAMA (triple combination) and ICS with LABA (dual combination) separately, anticipating increased underlying disease severity in people prescribed triple therapy. Second, we restricted analyses to the largest ethnic group (ie, white) to exclude any substantial confounding by ethnicity. We did not adjust for ethnicity in the main models because we did not anticipate this variable to be a strong confounder and due to a sizable proportion of individuals with missing ethnicity (23–24% across treatment categories). In the asthma population, we varied the sample definition to include people with asthma diagnosed at any time, and a prescription for any asthma medication within 4 months of the index date. In the COPD population, we included people who were prescribed LAMA monotherapy in the comparator group to investigate the effect of the choice to exclude this patient group in the main analyses. We also investigated additional adjustment for oral steroid use in the COPD population, for whom this variable did not form part of the exacerbation definition. Finally, post hoc, we re-ran the analyses using inverse probability of treatment-weighted Cox regressions, weighted using propensity scores derived from logistic (COPD) and multinomial logistic (asthma) models. Further details of these methods are in the appendix (p 30).

We hypothesised that respiratory disease severity, but not ICS use, might influence the risk of non-COVID-19-related death. Therefore, we did prespecified exploratory analyses using non-COVID-19-related death as a negative control outcome, censoring people at time of COVID-19-related death. If any potentially harmful association observed in primary analyses was due to confounding (ie, people prescribed ICS had more severe underlying respiratory disease than those who did not) we expected to observe a similar association with non-COVID-19-related death in people prescribed ICS.

Quantitative bias analysis is a type of sensitivity analysis that can be used to assess the role that unmeasured confounders might have in observational analyses. We used one form of quantitative bias analysis here specifically, we calculated an e value, which quantifies the strength of association between an unmeasured confounder and exposure or outcome, conditional on measured covariates, that would be necessary to fully explain observed associations.^37^

We managed the data using Python (version 3.8) and SQL, with analysis carried out using Stata (version 16.1). All code we used for data management and analyses is openly shared online on our GitHub page. All raw model outputs can be viewed online in the accompanying released output and log files that have had small numbers redacted. We followed both Strengthening the Reporting of Observational Studies in Epidemiology and Reporting of studies Conducted using Observational Routinely Collected Health Data Statement for Pharmacoepidemiology guidelines (appendix pp 40–43).

### Role of the funding source

The funder of the study had no role in the study design, data collection, data analysis, data interpretation, or writing of the report. AS, AJW, CEM, SB, WH, CB, JC, LS, and BG had access to the raw data. The corresponding author had full access to all the data in the study and had final responsibility for the decision to submit for publication.

## Results

Of 33 356 521 individuals in our linked database, 148 557 people with COPD and 818 490 people with asthma and a relevant prescription within the 4 months before the index date were eligible for inclusion in our two cohorts (appendix pp 3–4).

Demographic and clinical characteristics of the COPD population are shown in table 1. 43 308 (29·2%) of 148 557 individuals in this population had been given a LABA–LAMA prescription in the 4 months before the index date, and 105 249 (70·8%) had been given prescriptions for ICS–LABA or ICS–LABA–LAMA (ie, ICS combination). Demographic characteristics of treatment groups were similar. The median follow-up time was 66 days (IQR 66–66) in both groups (appendix p 39). The presence of comorbidities was similar between the two treatment groups, except asthma diagnosis in the 3 years before the index date, which was more common among people prescribed an ICS. The proportion of people with an exacerbation in the past year was lower among people prescribed a LABA–LAMA combination than those prescribed an ICS combination.

**Table 1:** Demographic and clinical characteristics of patients in the COPD cohort

		**LABA–LAMA combination (n=43 308)**	**ICS combination (n=105 249)**
**Demographics**			
Age, years			
	18–<40	85 (0·2%)	184 (0·2%)
	40–<50	1060 (2·5%)	2291 (2·2%)
	50–< 60	5749 (13·3%)	12 245 (11·6%)
	60–<70	12 607 (29·1%)	29 530 (28·1%)
	70–<80	16 106 (37·2%)	40 380 (38·4%)
	≥80	7701 (17·8%)	20 619 (19·6%)
	Median	71 (63–77)	72 (64–78)
	Mean	70·0 (10·1)	70·8 (10·1)
	Range	35–100	35–102
Sex			
	Female	19 717 (45·5%)	48 731 (46·3%)
	Male	23 591 (54·5%)	56 518 (53·7%)
BMI grouping			
	Underweight (<18·5 kg/m^2^)	1683 (3·9%)	4740 (4·5%)
	Normal (18·5–<25 kg/m^2^)	12 949 (29·9%)	31 865 (30·3%)
	Overweight (25–<30 kg/m^2^)	14 019 (32·4%)	33 514 (31·8%)
	Obese I (30–<35 kg/m^2^)	8593 (19·8%)	20 281 (19·3%)
	Obese II (35–<40 kg/m^2^)	3572 (8·3%)	8386 (8·0%)
	Obese III (≥40 kg/m^2^)	1581 (3·7%)	3948 (3·8%)
	Missing	911 (2·1%)	2515 (2·4%)
Smoking status			
	Never	0	0
	Former	26 040 (60·1%)	69 740 (66·3%)
	Current	17 268 (39·9%)	35 509 (33·7%)
	Missing	0	0
Ethnicity			
	White	32 498 (75·0%)	79 735 (75·8%)
	Mixed	92 (0·2%)	182 (0·2%)
	Asian or Asian British	260 (0·6%)	836 (0·8%)
	Black	103 (0·2%)	301 (0·3%)
	Other	112 (0·3%)	289 (0·3%)
	Unknown	10 243 (23·7%)	23 906 (22·7%)
Index of Multiple Deprivation			
	1 (least deprived)	8066 (18·6%)	19 896 (18·9%)
	2	8426 (19·5%)	20 629 (19·6%)
	3	8757 (20·2%)	21 244 (20·2%)
	4	8425 (19·5%)	21 641 (20·6%)
	5 (most deprived)	9634 (22·3%)	21 839 (20·%)
	Missing	0	0
**Treatments**			
Single SABA		30 945 (71·5%)	83 683 (79·5%)
High-dose ICS		0	25 467 (24·2%)
Low-dose or medium-dose ICS		0	82 506 (78·4%)
Single ICS		0	2325 (2·2%)
Single SAMA		157 (0·4%)	1488 (1·4%)
Single LABA		2433 (5·6%)	855 (0·8%)
Single LAMA		4590 (10·6%)	45 483 (43·2%)
LABA–ICS		0	75 552 (71·8%)
LABA–LAMA		41 377 (95·5%)	4807 (4·6%)
LABA–LAMA–ICS		0	33 040 (31·4%)
Single LTRA		0	0
**Clinical conditions**			
Chronic kidney disease			
	No	35 570 (82·1%)	86 886 (82·6%)
	Yes	7738 (17·9%)	18 363 (17·4%)
Hypertension			
	No	21 607 (49·9%)	50 954 (48·4%)
	Yes	21 701 (50·1%)	54 295 (51·6%)
Heart failure			
	No	39 445 (91·1%)	95 283 (90·5%)
	Yes	3863 (8·9%)	9966 (9·5%)
Other heart diseases			
	No	33 253 (76·8%)	81 128 (77·1%)
	Yes	10 055 (23·2%)	24 121 (22·9%)
Cancer			
	No	37 073 (85·6%)	90 171 (85·7%)
	Yes	6235 (14·4%)	15 078 (14·3%)
Diabetes			
	No diabetes	32 913 (76·0%)	79 549 (75·6%)
	Diabetes, not severe	7586 (17·5%)	19 030 (18·1%)
	Diabetes, severe	2712 (6·3%)	6 366 (6·0%)
	Diabetes, no HbA_1C_ measurement	97 (0·2%)	304 (0·3%)
Recent statin use			
	No	20 531 (47·4%)	50 912 (48·4%)
	Yes	22 777 (52·6%)	54 337 (51·6%)
Influenza vaccine status			
	No	8689 (20·1%)	19 917 (18·9%)
	Yes	34 619 (79·9%)	85 332 (81·1%)
Pneumococcal vaccine status			
	No	32 285 (74·5%)	83 943 (79·8%)
	Yes	11 023 (25·5%)	21 306 (20·2%)
Exacerbation in past year			
	No	34 774 (80·3%)	77 897 (74·0%)
	Yes	8534 (19·7%)	27 352 (26·0%)
Asthma ever*			
	No	37 731 (87·1%)	76 063 (72·3%)
	Yes	5577 (12·9%)	29 186 (27·7%)
Immunosuppressed (combination algorithm)			
	No	43 211 (99·8%)	105 013 (99·8%)
	Yes	97 (0·2%)	236 (0·2%)
Primary care physician consultation count			
	Median	10 (6–16)	10 (6–17)
	Mean	12·7 (11·4)	13·5 (12·3)
	Range	0–276	0–306
Exacerbation count in the past year			
	Median	0 (0–0)	0 (0–1)
	Mean	0·26 (0·60)	0·38 (0·77)
	Range	0–9	0–12

Data are n (%), median (IQR), mean (SD), or range. Some proportions might not add up to 100% due to rounding. BMI=body-mass index. COPD=chronic obstructive pulmonary disease. HbA_1c_=glycated haemoglobin. ICS=inhaled corticosteroid. LABA=long-acting β agonist. LAMA=long-acting muscarinic antagonist. LTRA=leukotriene receptor antagonist. SABA=short-acting β agonist. SAMA=short-acting muscarinic antagonist.

* Asthma diagnosed >3 years before index date.

Demographic and clinical characteristics of the asthma population are shown in table 2. 608 972 (74·4%) of 818 490 individuals had a prescription for low-dose or medium-dose ICS in the 4 months before the index date, 101 077 (12·3%) had a prescription for a high-dose ICS, and 108 441 (13·2%) had a prescription for a SABA only. The asthma treatment groups differed in terms of demographic and clinical characteristics. The median age was 48 years (IQR 35–60) in the SABA only group, 53 years (40–66) in the low-dose or medium-dose ICS group, and 55 years (44–67) in the high-dose ICS group. The proportion of men was slightly higher among people prescribed SABA only (46 614 [43·0%]) than in the low-dose or medium-dose ICS group (245 446 [40·3%]) and high-dose ICS group (38 590 [38·2%]). The median follow-up time was 66 days (IQR 66–66) in all groups. The prevalence of most comorbidities was lowest among people prescribed SABA only and highest among those prescribed high-dose ICS. The SABA only group had the lowest proportion of individuals with an asthma exacerbation in the past year (15 210 [14·0%]), and the high-dose ICS group had the highest proportion (36 726 [36·3%]).

**Table 2:** Demographic and clinical characteristics for patients in asthma cohort

		**SABA only (n=108 411)**	**ICS**	
			Low or medium dose (n=608 972)	High dose (n=101 077)
**Demographics**				
Age				
	18–<40	36 264 (33·4%)	144 955 (23·8%)	18 836 (18·6%)
	40–<50	22 067 (20·4%)	107 835 (17·7%)	18 459 (18·3%)
	50–< 60	21 852 (20·2%)	130 434 (21·4%)	23 752 (23·5%)
	60–<70	13 974 (12·9%)	105 897 (17·4%)	18 970 (18·8%)
	70–<80	9209 (8·5%)	79 810 (13·1%)	13 904 (13·8%)
	≥80	5075 (4·7%)	40 041 (6·6%)	7156 (7·1%)
	Median	48 (35–60)	53 (40–66)	55 (44–67)
	Mean	48·3 (17·4)	53·1 (17·4)	55·0 (16·4)
	Range	18–106	18–106	18–106
Sex				
	Female	61 827 (57·0%)	363 526 (59·7%)	62 487 (61·8%)
	Male	46 614 (43·0%)	245 446 (40·3%)	38 590 (38·2%)
BMI grouping				
	Underweight (<18·5 kg/m^2^)	1637 (1·5%)	7623 (1·3%)	1147 (1·1%)
	Normal (18·5–<25 kg/m^2^)	28 141 (26·0%)	153 022 (25·1%)	20 955 (20·7%)
	Overweight (25–<30 kg/m^2^)	32 874 (30·3%)	196 599 (32·3%)	30 763 (30·4%)
	Obese I (30–<35 kg/m^2^)	20 069 (18·5%)	122 212 (20·1%)	22 297 (22·1%)
	Obese II (35–<40 kg/m^2^)	9497 (8·8%)	56 837 (9·3%)	11 891 (11·8%)
	Obese III (≥40 kg/m^2^)	5937 (5·5%)	34 102 (5·6%)	8248 (8·2%)
	Missing	10 286 (9·5%)	38 577 (6·3%)	5776 (5·7%)
Smoking status				
	Never	45 384 (41·9%)	268 922 (44·2%)	41 231 (40·8%)
	Former	42 272 (39·0%)	254 334 (41·8%)	44 137 (43·7%)
	Current	20 625 (19·0%)	85 414 (14·0%)	15 665 (15·5%)
	Missing	160 (0·2%)	302 (0·1%)	44 (<0·1%)
Ethnicity				
	White	74 402 (68·6%)	428 142 (70·3%)	71 303 (70·5%)
	Mixed	978 (0·9%)	5028 (0·8%)	838 (0·8%)
	Asian or Asian British	5698 (5·3%)	32 357 (5·3%)	5866 (5·8%)
	Black	1546 (1·4%)	8139 (1·3%)	1455 (1·4%)
	Other	892 (0·8%)	4863 (0·8%)	881 (0·9%)
	Unknown	24 925 (23·0%)	130 443 (21·4%)	20 734 (20·5%)
Index of Multiple Deprivation				
	1 (least deprived)	21 087 (19·5%)	124 406 (20·4%)	17 776 (17·6%)
	2	21 705 (20·0%)	124 312 (20·4%)	18 956 (18·8%)
	3	22 016 (20·3%)	121 926 (20·0%)	20 217 (20·0%)
	4	22 392 (20·7%)	121 117 (19·9%)	21 478 (21·2%)
	5 (most deprived)	21 241 (19·6%)	117 211 (19·3%)	22 650 (22·4%)
	Missing	0	0	0
**Treatments**				
Single SABA		108 441 (100%)	417 008 (68·5%)	78 222 (77·4%)
High-dose ICS		0	2914 (0·5%)	101 077 (100%)
Low-dose or medium-dose ICS		0	608 972 (100%)	7547 (7·5%)
Single ICS		0	280 880 (46·1%)	10 088 (10·0%)
Single SAMA		116 (0·1%)	1630 (0·3%)	896 (0·9%)
Single LABA		543 (0·5%)	5960 (1·0%)	1503 (1·5%)
Single LAMA		0	8037 (1·3%)	8335 (8·3%)
LABA–ICS		0	338 182 (55·5%)	93 459 (92·5%)
LABA–LAMA		0	236 (<0·1%)	121 (0·1%)
LABA–LAMA–ICS		0	1177 (0·2%)	86 (0·1%)
Single LTRA		0	40 468 (6·7%)	23 054 (22·8%)
**Clinical conditions**				
Chronic kidney disease				
	No	103 341 (95·3%)	573 525 (94·2%)	94 026 (93·0%)
	Yes	5100 (4·7%)	35 447 (5·8%)	7051 (7·0%)
Hypertension				
	No	82 598 (76·2%)	432 452 (71·0%)	67 206 (66·5%)
	Yes	25 843 (23·8%)	176 520 (29·0%)	33 871 (33·5%)
Heart failure				
	No	106 459 (98·2%)	596 359 (97·9%)	98 081 (97·0%)
	Yes	1982 (1·8%)	12 613 (2·1%)	2996 (3·0%)
Other heart diseases				
	No	101 549 (93·7%)	565 066 (92·8%)	91 929 (90·9%)
	Yes	6892 (6·4%)	43 906 (7·2%)	9148 (9·1%)
Cancer				
	No	102 740 (94·7%)	571 390 (93·8%)	94 325 (93·3%)
	Yes	5701 (5·3%)	37 582 (6·2%)	6752 (6·7%)
Diabetes				
	No diabetes	93 589 (86·3%)	523 874 (86·0%)	83 064 (82·2%)
	Diabetes, not severe	8946 (8·3%)	57 866 (9·5%)	12 254 (12·1%)
	Diabetes, severe	5 551 (5·1%)	25 283 (4·2%)	5436 (5·4%)
	Diabetes, no HbA_1c_	355 (0·3%)	1949 (0·3%)	323 (0·3%)
Recent statin use				
	No	90 340 (83·3%)	475 969 (78·2%)	74 854 (74·1%)
	Yes	18 101 (16·7%)	133 003 (21·8%)	26 223 (25·9%)
Influenza vaccine status				
	No	65 191 (60·1%)	240 861 (39·6%)	36 016 (35·6%)
	Yes	43 250 (39·9%)	368 111 (60·5%)	65 061 (64·4%)
Pneumococcal vaccine status				
	No	101 846 (93·9%)	557 804 (91·6%)	91 105 (90·1%)
	Yes	6595 (6·1%)	51 168 (8·4%)	9972 (9·9%)
Exacerbation in past year				
	No	93 231 (86·0%)	487 282 (80·0%)	64 351 (63·7%)
	Yes	15 210 (14·0%)	121 690 (20·0%)	36 726 (36·3%)
Immunosuppressed (combination algorithm)				
	No	108 056 (99·6%)	607 221 (99·7%)	100 762 (99·7%)
	Yes	385 (0·4%)	1751 (0·3%)	315 (0·3%)
Primary care physician consultation count				
	Median	6 (3–12)	7 (4–13)	9 (5–16)
	Mean	9·3 (10·7)	10·3 (11·2)	12·8 (13·5)
	Range	0–296	0–548	0–604
Exacerbation count in the past year				
	Median	0 (0–0)	0 (0–0)	0 (0–1)
	Mean	0·24 (0·95)	0·35 (1·09)	0·81 (1·71)
	Range	0–16	0–17	0–17

Data are n (%), median (IQR), mean (SD), or range. Some proportions might not add up to 100% due to rounding. BMI=body-mass index. HbA_1c_=glycated haemoglobin. ICS=inhaled corticosteroid. LABA=long-acting β-agonist. LAMA=long-acting muscarinic antagonist. LTRA=leukotriene receptor antagonist. SABA=short-acting β agonist. SAMA=short-acting muscarinic antagonist.

**Figure 1:**
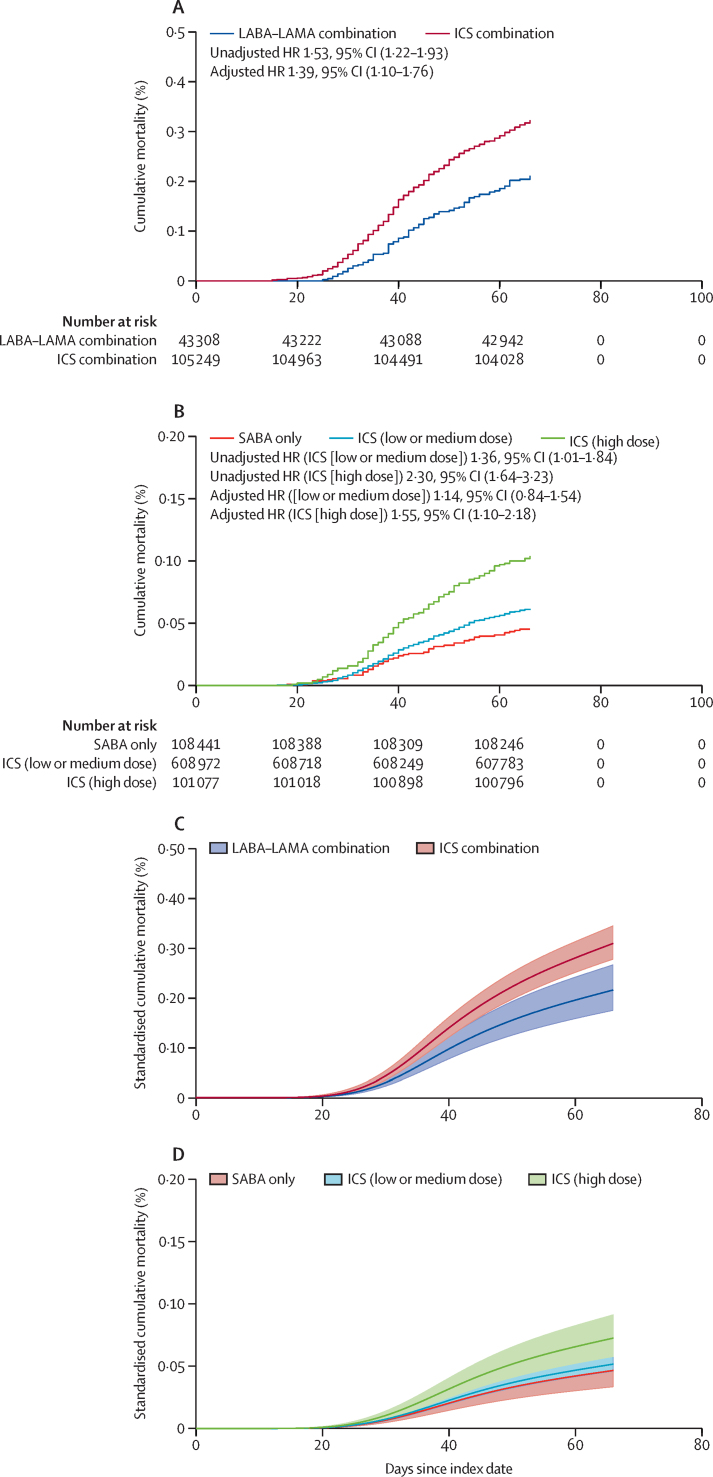
Time to COVID-19-related death for the COPD population (A) and asthma population (B), and standardised survival curves for the COPD (C) and asthma (D) populations In panels C and D, the solid line shows the standardised cumulative mortality, and the shaded area the 95% CI. COPD=chronic obstructive pulmonary disease. HR=hazard ratio. ICS=inhaled corticosteroid. LABA=long-acting β agonist. LAMA=long-acting muscarinic antagonist. SABA=short-acting β agonist.

**Figure 2:**
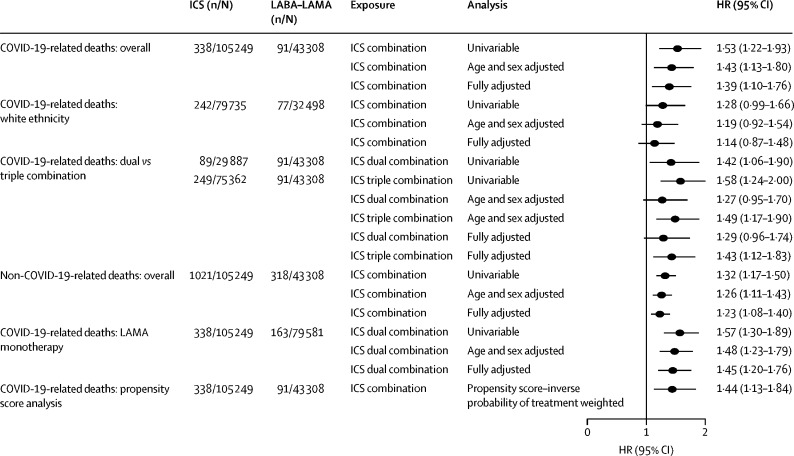
Forest plot of COVID-19-related deaths in the COPD population, overall and sensitivity analyses COPD=chronic obstructive pulmonary disease. HR=hazard ratio. ICS=inhaled cortecosteroid. LABA=long-acting β agonist. LAMA=long-acting muscarinic antagonist.

429 COVID-19-related deaths occurred in the COPD population. Time to COVID-19-related death by treatment group is shown in figure 1A. In univariable models, compared with people prescribed LABA–LAMA combinations, those prescribed ICS combinations had an increased risk of COVID-19-related death (HR 1·53 [95% CI 1·22–1·93]; figure 2). This association decreased after adjustment for age and sex (1·43 [1·13–1·80]), and in the fully adjusted model (1·39 [1·10–1·76]; figure 2). Post-hoc analyses showed that adjustment for previous exacerbations as a binary variable, smoking status, and index of multiple deprivation had the largest effect on reducing the association; adjustment for other individual potential confounders had a negligible effect on the effect estimate (appendix pp 17–18). We found no evidence of a (prespecified) interaction with age (appendix p 14). We detected some evidence of deviations from the proportional hazards assumptions (appendix pp 15–16). The Kaplan-Meier curve indicated that the HR was probably above 1 throughout the follow-up period, with the effect size growing slightly over time (figure 1A).

We generated standardised survival curves for the COPD population (figure 1C). At the end of follow-up, estimated cumulative mortality was 0·31% (95% CI 0·28–0·35) in the ICS combination group and 0·22% (0·17–0·27) in the LABA–LAMA combination group. The adjusted absolute cumulative risk difference between the two treatment groups at the end of follow-up was 0·09% (0·03–0·15).

**Figure 3:**
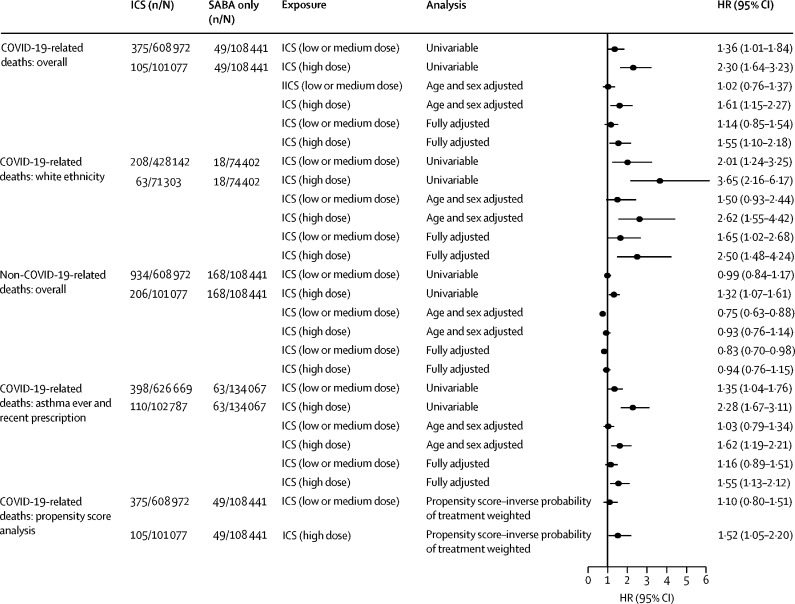
Forest pot of COVID-19-related deaths in the asthma population, overall and sensitivity analyses HR=hazard ratio. ICS=inhaled cortecosteroid. SABA=short-acting β agonist.

529 COVID-19-related deaths occurred in the asthma population. Time to COVID-19-related death by treatment group is shown in figure 1B. In univariable models, people prescribed both low-dose or medium-dose ICS and high-dose ICS were at an increased risk of COVID-19-related death compared with those prescribed SABA only (HR 1·36 [95% CI 1·01–1·84] for low or medium dose; 2·30 [1·64–3·23] for high dose; figure 3). These associations reduced substantially after adjustment for age and sex (1·02 [0·76–1·37] for low or medium dose; 1·61 [1·15–2·27] for high dose), and in fully adjusted models (1·14 [0·85–1·54] for low or medium dose; 1·55 [1·10–2·18] for high dose; figure 3). Post-hoc analyses revealed that the greatest reduction in the strength of the association between high-dose ICS and COVID-19-related mortality, after adjustment for age and sex, was adjustment for previous exacerbations; adjustment for other individual potential confounders had a negligible effect on the effect estimate (appendix p 25–26). We found no evidence for a (prespecified) interaction with age, and no deviations from the proportional hazards assumption (appendix pp 20–24).

We generated standardised survival curves for the asthma population (figure 1D). At the end of follow-up, the estimated cumulative mortality was 0·07% (95%CI 0·06 to 0·09) in the high-dose ICS group, 0·05% (0·05 to 0·06) in the low-dose ICS group, and 0·05% (0·03 to 0·07) in the SABA only group. The adjusted absolute cumulative risk difference was 0·005% (−0·0012 to 0·002) comparing the low-dose or medium-dose ICS group with the SABA only group and 0·03% (0·003 to 0·05) comparing the high-dose ICS group to the SABA only group.

In our sensitivity analyses, when considering the dual combination of ICS–LABA and the triple combination of ICS–LABA–LAMA separately in the COPD population compared with the main analysis, the risk of death was increased among those prescribed triple combination therapy (fully adjusted HR 1·43 [95% CI 1·12–1·83]) but this increase was less substantial among those prescribed dual combination therapy (1·29 [0·96–1·74]; figure 2). Restricting analyses to people of white ethnicity led to a reduction in the HRs in the COPD population (figure 2), but not in the asthma population (figure 3). Changing the population definition for the asthma population had a negligible effect on the results (figure 3). Including people receiving LAMA monotherapy in the COPD comparator group also did not substantially affect the main effect estimates (figure 2), and additional adjustment for oral steroid use in the COPD population when this did not form part of the exacerbation definition also did not affect the effect estimate (appendix pp 18). Finally, re-running the analyses using propensity scores incorporated through inverse probability of treatment-weighted Cox regressions did not change the effect estimates (Figure 2, Figure 3; appendix p 38).

In the COPD population, the risk of non-COVID-19-related death was higher among individuals prescribed ICS than in those prescribed LABA–LAMA with an adjusted HR of 1·23 (95% CI 1·08–1·40; figure 2). In the asthma population, we found no evidence of an increased risk of non-COVID-19-related death among individuals prescribed high-dose or low-dose or medium-dose ICS compared with those prescribed SABA only (figure 3).

Given that we observed a harmful association between ICS and COVID-19 death, we used quantitative bias analysis to see how much unadjusted confounding would need to be present in our data to account for this result, if in reality, the risk of COVID-19-related death is not affected by ICS use. To make our results consistent with a null effect—ie, to fully explain the lower bound of the fully adjusted 95% CI in the COPD cohort (1·10), or for the high-dose ICS association in the asthma cohort (1·10)—an unmeasured confounder would need to be associated (conditional on measured covariates) with either exposure or outcome by a risk ratio of at least 1·43 (e value; appendix p 28). An unmeasured confounder would need to have a stronger association with either exposure or outcome to move the observed HRs to a protective effect of 0·8 (e value COPD: 2·87, asthma: 3·29, appendix p 29).

## Discussion

We have investigated the association between regular ICS use and COVID-19-related death among people with chronic respiratory disease in England. In a cohort of people with COPD, the HR for COVID-19-related death among those prescribed ICS combinations compared with people prescribed LABA–LAMA combinations was 1·39 (95% CI 1·10–1·76) in the fully adjusted model, although this statistic does not mean the difference was caused by their ICS prescription. In the asthma cohort, those prescribed high-dose ICS had an HR for COVID-19-related death of 1·55 (95% CI 1·10–2·18) compared with those prescribed SABA only in the fully adjusted model, with little evidence of any increased relative risk of death among those prescribed low-dose or medium-dose ICS (1·14 [0·85–1·54]). Notably, absolute risks of death were very low, with the estimated cumulative COVID-19-related mortality only 0·09% higher in the ICS group than in the LABA–LAMA group in the COPD population, and 0·03% higher in the high-dose ICS group than in the SABA only group in the asthma population. Taken together, our findings do not provide any strong support for a protective effect from ICS use in these populations, as has been previously hypothesised might exist.

The observed harmful associations between ICS prescription and COVID-19-related death could be readily explained by confounding due to underlying health differences between people prescribed ICS and those using other medications for asthma and COPD, differences that cannot be measured using EHR data, rather than representing a causally harmful effect of ICS. In support of this interpretation, we observed a higher risk of COVID-19-related death among people prescribed ICS triple therapy than in those prescribed dual therapy in the COPD cohort. The ICS content of these two regimens is similar, and any putative causal effect of ICS would be expected to be similar in these two groups. If it had been possible to successfully control for differences in disease severity between treatment groups, we would also expect to see no increased risk of death in the ICS groups for the negative control outcome of non-COVID-19-related death. The harmful association between ICS use and non-COVID-19-related death we observed suggests we were not able to capture all markers of disease severity using patients' EHR data, resulting in an observed association that is unlikely to be causal. The absence of an increased risk of non-COVID-19-related death among people prescribed ICS combinations in the asthma cohort is perhaps not surprising because the association between asthma and overall mortality is much smaller than that for COPD.^38^ Finally, quantitative bias analysis confirmed that a hypothetical unmeasured confounder of moderate strength could fully explain the observed results.

Notably, had ICS been responsible for the protective effect alluded to in previous descriptive studies,^8^, ^10^, ^11^ we would expect to detect such an association in our study irrespective of unmeasured confounding by indication. We would expect to see this association because these descriptive studies have compared the risk of severe COVID-19 outcomes among patients with respiratory disease, only some of whom use ICSs, to those without any respiratory disease at all^8^ or those with only mild disease.^11^ To explain any reduced risk in these studies, the effect of ICS use would have to be strong enough to overcome both unmeasured confounding due to differences in respiratory disease severity and a dilution of effect because not all patients will have been prescribed ICSs. Therefore, although unmeasured confounding is a plausible explanation for the harmful effect we observed, it cannot fully explain the absence of a protective effect of the size that would have to exist according to the original hypothesis.

To our knowledge, no other epidemiological studies or randomised controlled trials assessing the role of regular ICS use in COVID-19 among people with an indication have been done to date. Two randomised trials investigating the role of ICS use in people who have been admitted to hospital with laboratory-confirmed SARS-CoV-2 infection (NCT04331054) and mild COVID-19 (NCT04330586) are ongoing. The hypothesis of a protective effect of ICS in COVID-19 was partly based on the low prevalence of chronic lung disease among outpatient and inpatient COVID-19 cases in China.^8^ However, subsequent studies do not support initial assertions that people with chronic lung diseases (including COPD) are substantially under-represented among patients with COVID-19.^5^, ^6^, ^39^ Additionally, a few studies have found that people with COPD are at increased risk of severe COVID-19 and death from COVID-19.^5^, ^6^ The evidence for an association between asthma and COVID-19 severity is more varied, with studies reporting both null and moderately harmful associations.^5^, ^6^ Among people with asthma, features other than ICS use (eg, shielding) might affect the risk of acquiring SARS-CoV-2. Studies investigating the causal effect of chronic respiratory disease, including COPD and asthma, on the risk of SARS-CoV-2 infection and COVID-19 severity, ideally taking into account the relatively large degree of heterogeneity that exists in each of these diagnostic categories, are urgently needed to help inform decisions around levels of risk for these individuals.

The greatest strength of this study was our ability to look at multiple drug treatments because our dataset included medical records from more than 30 million individuals. Our study is further strengthened by our use of two different study populations and active comparators, and sensitivity analyses to quantify the potential effect of unmeasured confounding on results. Finally, we used open methods: we prespecified our analysis plan and have shared all analytical code.

Our study has several limitations. The primary limitation is the risk of confounding by indication due to unmeasured or imperfectly defined potential confounding variables. Decisions regarding treatment choices involve factors that might not be well recorded in EHRs, including measures such as spirometry and probable steroid responsiveness. Other important unmeasured markers of severity include the use of home oxygen therapy, which is typically not issued as a prescription by English primary care physicians. Because we did not have secondary care data, our assessment of exacerbation history was incomplete, restricting our ability to adjust for this variable. Our sensitivity analyses suggest that moderate unmeasured confounding is a plausible reason for the harmful associations we observed. Another important consideration is the risk of exposure misclassification. Some patients might have been incorrectly classified as being exposed to ICS, when in reality they were not using these medications. Using prescription frequency before the English lockdown (which started on March 23, 2020) as a proxy for adherence to identify patients who are more likely to be regular users would probably be relatively inaccurate, both due to the risk of as-needed use of some inhalers and the stockpiling of inhalers before the start of the UK lockdown.^40^ We found some evidence that the proportional hazards assumption was not met for the COPD models, with Kaplan-Meier plots indicating that the HR for exposure to ICS versus LABA–LAMA increased over time. This observation is perhaps not surprising, because the risk of acquiring COVID-19 was lower in early March, 2020, before lockdown, when SARS-CoV-2 was more widespread. The HR for risk of COVID-19-related death in the COPD population should be interpreted as an average over the entire follow-up period. Finally, the outcome of COVID-19-related death will reflect the risk both of becoming infected with SARS-CoV-2 and the risk of developing severe disease and dying. ICS use might have a different effect on the risk of infection and on disease severity. We were not able to explore the relative contribution of ICS use on each of these endpoints in our study because of the absence of complete or representative data on rates of infection with SARS-CoV-2 in the UK. Test result datasets are not an adequate proxy for these data because tests have largely been restricted to patients who already have relatively severe disease and so are not representative of rates of infection in the general population. This situation might change as testing capacity expands and other studies might be able to test these hypotheses in the future. However, if ICS use had a strong protective effect on the risk of SARS-CoV-2 infection, we would expect to see this association via a reduced rate of COVID-19-related mortality compared with people given alternative treatments because infection is considerably more common than death. In terms of generalisability, we did not expect the pharmacological effect of ICS use on COVID-19-related outcomes to vary substantially according to the distribution of population characteristics; however, because this effect has not been widely studied to date, our findings might not be generalisable to other settings or populations. Finally, this study has addressed whether people prescribed ICS for use in asthma or COPD had reduced COVID-19-related mortality compared with those prescribed other medications during the current SARS-CoV-2 outbreak. We have not assessed the role of ICS use in treating COVID-19 among patients without asthma or COPD; ongoing trials will provide evidence on this matter (NCT04331054, NCT04330586).

The totality of the data presented here, including our sensitivity analyses, do not indicate that regular ICS therapy for asthma or COPD either decreases or increases risk of death from COVID-19, and do not provide evidence to support adjustments in ICS therapy among patients with asthma or COPD during outbreaks of SARS-CoV-2. The absence of evidence for a protective effect implies that alternative explanations for the observed under-representation of chronic respiratory disease in early epidemiological studies of patients with COVID-19 should be explored in future studies. Because the small observed increased risk of death is unlikely to be causal, our results should also provide reassurance to patients relying on ICSs that the use of these medications have not put them at undue risk of negative outcomes during the ongoing pandemic. Finally, we note that future observational studies of this clinical question are likely to face similar challenges around unmeasured confounding as those described here and we encourage researchers to undertake appropriate sensitivity analyses where possible.

The UK has an unusually large volume of detailed longitudinal patient data. We have shown that it is feasible to rapidly address specific hypotheses about medicines in a transparent manner inside the secure environment of an EHR vendor to minimise the large volumes of potentially disclosive data that would otherwise have to move into separate systems. We will use the OpenSAFELY platform to further inform the global response about drug treatments during the COVID-19 emergency.

In summary, we found no evidence of a beneficial effect of regular ICS use among people with COPD and asthma on COVID-19-related mortality. Although we report a small harmful association, the pattern of results we observed suggests this association could readily be explained by differences in underlying health between people prescribed ICSs and those prescribed other respiratory medications. These results do not support any change to the current clinical guidelines for the routine treatment of people with COPD or asthma with ICS during outbreaks of SARS-CoV-2 infection.

For the **Johns Hopkins Coronavirus Resource Center** see https://coronavirus.jhu.edu/map.html

For the **OpenSAFELY website** see https://opensafely.org

For the **study protocol** see https://github.com/opensafely/ics-research/tree/master/protocol

For a **full list of codelists** see https://codelists.opensafely.org

For our **GitHub page** see https://github.com/opensafely/ics-research

For **output and log files** see https://github.com/opensafely/ics-research/tree/master/released_analysis_results

For the **OpenPrescribing website** see https://openprescribing.net/

## Data sharing

All data were linked, stored, and analysed securely within the OpenSAFELY platform. Detailed pseudonymised patient data are potentially re-identifiable and therefore are not shared. We rapidly delivered the OpenSAFELY data analysis platform without prior funding to deliver timely analyses on urgent research questions in the context of the global COVID-19 health emergency. Now that the platform is established, we are developing a formal process for external users to request access in collaboration with NHS England. Details of this process should be published shortly on the OpenSAFELY website.
