# Supplementary material for: Risk of COVID-19-related death among patients with chronic obstructive pulmonary disease or asthma prescribed inhaled corticosteroids: an observational cohort study using the OpenSAFELY platform
Source: Lancet Respir Med. 2020 Nov;8(11):1106–20. doi: 10.1016/S2213-2600(20)30415-X (PMC7515601; doi:10.1016/S2213-2600(20)30415-X)
Supplement: Supplementary appendix [file mmc1.pdf]

THE LANCET
Respiratory Medicine

Supplementary appendix

This appendix formed part of the original submission. We post it as supplied by the authors.

Supplement to: Schultze A, Walker AJ, MacKenna B, et al. Risk of COVID-19-related death among patients with chronic obstructive pulmonary disease or asthma prescribed inhaled corticosteroids: an observational cohort study using the OpenSAFELY platform. *Lancet Respir Med* 2020; September 24, 2020. https://doi.org/10.1016/S2213-2600(20)30415-X.

Supplemental Materials

Paper: Risk of COVID-19-related death among patients with chronic obstructive pulmonary disease or asthma prescribed inhaled corticosteroids: an observational cohort study using the OpenSAFELY platform

Date: 21 September 2020

Please note: *Content is formatted for easy display and to match journal submission requirements. The raw results outputs can be found on our github repo under released_analysis_results (with/without date release)*

Table of Contents

Table 1. Demographic and Clinical Characteristics - COPD 5
Table 2. Demographic and Clinical Characteristics - Asthma 9
Table 3. Association between current ICS use and COVID-19 Death* in ONS – All COPD analyses 13
Table 4. Current ICS use and COVID-19 Death in ONS, Age Interaction - COPD Population 14
Table 5. Testing the PH assumption for COVID-19 Death in ONS- COPD Population 15
Table 6. 1 by 1 comorbidity adjustments (after age/sex and strata adjustments) - COPD population 17
Table 7. Additional adjustments - COPD 18
Table 8. Association between current ICS use and COVID-19 Death* in ONS – All Asthma Analyses 19
Table 9. Current ICS use and COVID-19 Death in ONS, Age Interaction - Asthma Population 20
Table 10. Testing the PH assumption for COVID-19 Death in ONS - Asthma Population 21
Table 11. 1 by 1 comorbidity adjustments (after age/sex and strata adjustments) - Asthma population 25
Table 12. Additional adjustments - asthma 27
Table 13. Standardised Differences Before and After Weighting in the COPD population 30
Table 14. Standardised Differences Before and After Weighting in the Asthma population 31
Table 15. Association between ICS and COVID-19 deaths in the COPD population - IPTW models 38
Table 16. Association between ICS and COVID-19 deaths in the Asthma population - IPTW models 38
Table 17. Summary of available follow-up time, by treatment group in the COPD population 39
Table 18. Summary of available follow-up time, by treatment group in the asthma population 39
Table 19. STROBE and RECORD checklist 40

Figure 1. Illustration of the Study Design 2
Figure 2. Flowchart - COPD population 3
Figure 3. Flowchart - Asthma Population 4
Figure 4. Schoenfeld residuals, unadjusted 15
Figure 5. Schoenfeld residuals, age/sex adjusted 16
Figure 6. Schoenfeld residuals, fully adjusted 16
Figure 7. Schoenfeld residuals, unadjusted ICS (low/medium-dose) 21
Figure 8. Schoenfeld residuals, age/sex adjusted ICS (low/medium dose) 22
Figure 9. Schoenfeld residuals, fully adjusted ICS (low/medium dose) 22
Figure 10. Schoenfeld residuals, unadjusted ICS (high-dose) 23
Figure 11. Schoenfeld residuals, age/sex adjusted ICS (high-dose) 23
Figure 12. Schoenfeld residuals, fully adjusted ICS (high-dose) 24
Figure 13. E-value for the lower 95% CI and point estimate for the ICS COPD association, assuming the true effect is 1.0 28
Figure 14. E-value for the lower 95% CI and point estimate for the high-dose ICS asthma association, assuming the true effect is 0.8 28
Figure 15. E-value for the lower 95% CI and point estimate for the ICS COPD association, assuming the true effect is 0.8 29
Figure 16. E-value for the lower 95% CI and point estimate for the high-dose ICS asthma association, assuming the true effect is 0.8 29
Figure 17. COPD: Kernel Density Plot of PS by treatment, before weighting 32
Figure 18. COPD: Kernel Density Plot of PS by treatment, after weighting (ATE) 32
Figure 19. COPD: Kernel Density Plot of PS by treatment, after weighting (ATT) 33
Figure 20. Asthma: Kernel Density Plot of Propensity Score for SABA only by treatment, before weighting 33
Figure 21. Asthma: Kernel Density Plot of Propensity Score for SABA only by treatment, after weighting (ATE) 34
Figure 22. Asthma: Kernel Density Plot of Propensity Score for SABA only by treatment, after weighting (ATT) 34
Figure 23. Asthma: Kernel Density Plot of Propensity Score for Low/Medium Dose ICS by treatment, before weighting 35
Figure 24. Asthma: Kernel Density Plot of Propensity Score for Low/Medium Dose ICS by treatment, after weighting (ATE) 35
Figure 25.Asthma: Kernel Density Plot of Propensity Score for Low/Medium Dose ICS by treatment, after weighting (ATT) 36
Figure 26. Asthma: Kernel Density Plot of Propensity Score for High Dose ICS by treatment, before weighting 36
Figure 27. Asthma: Kernel Density Plot of Propensity Score for High Dose ICS by treatment, after weighting (ATE) 37
Figure 28. Asthma: Kernel Density Plot of Propensity Score for High Dose ICS by treatment, after weighting (ATT) 37

Information Governance 44

Figure 1. Illustration of the Study Design

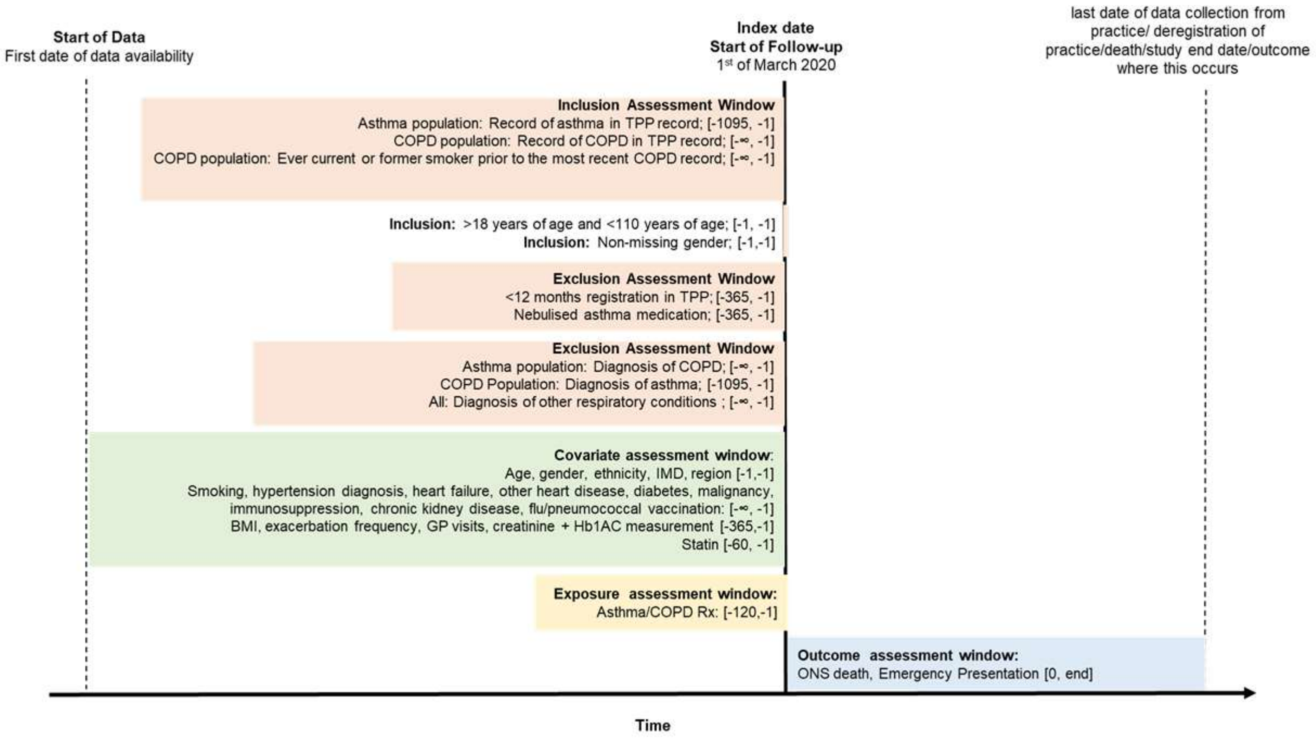

Figure 2. Flowchart - COPD population

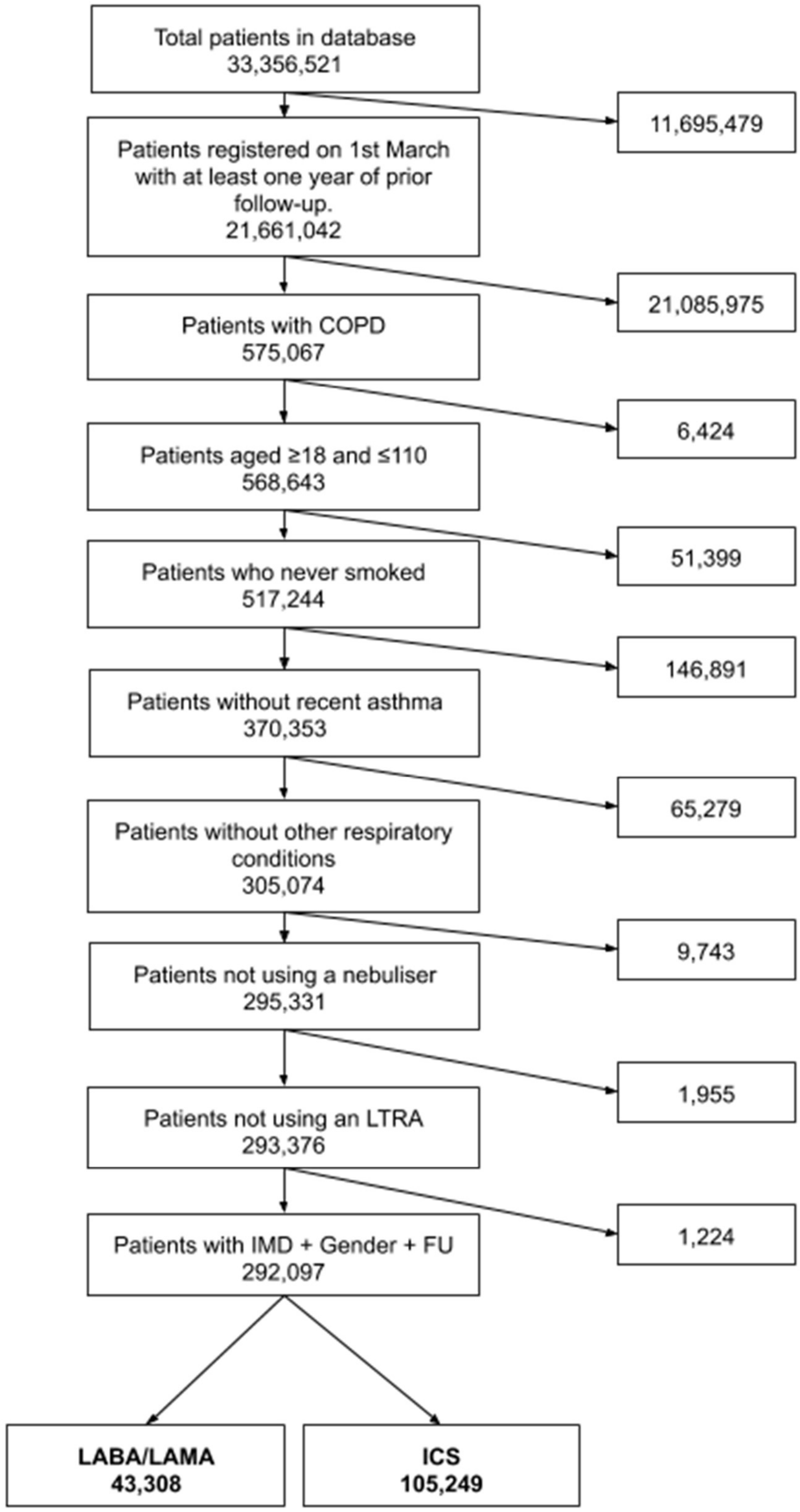

Figure 3. Flowchart - Asthma Population

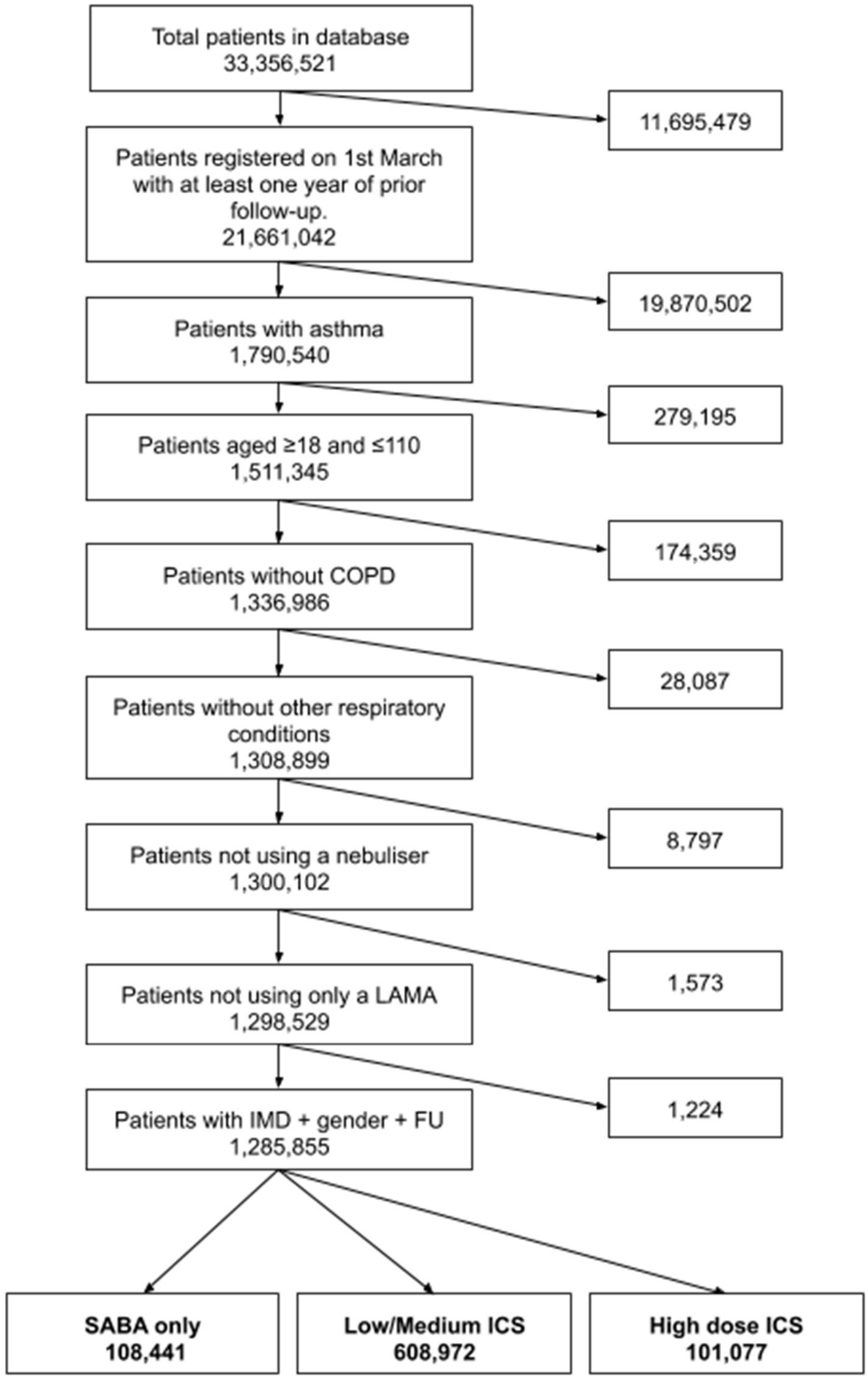

Table 1. Demographic and Clinical Characteristics - COPD

		Total	LABA/LAMA Combination	ICS Combination	Other
**Total Included**		**292,097 (100.0)**	**43,308 (100.0)**	**105,249 (100.0)**	**143,540 (100.0)**
**Demographics**					
Grouped age	18 - <40	1,235 (0.4)	85 (0.2)	184 (0.2)	966 (0.7)
	40 - <50	10,046 (3.4)	1,060 (2.4)	2,291 (2.2)	6,695 (4.7)
	50 - < 60	40,956 (14.0)	5,749 (13.3)	12,245 (11.6)	22,962 (16.0)
	60 - <70	80,874 (27.7)	12,607 (29.1)	29,530 (28.1)	38,737 (27.0)
	70 - <80	104,405 (35.7)	16,106 (37.2)	40,380 (38.4)	47,919 (33.4)
	80+	54,581 (18.7)	7,701 (17.8)	20,619 (19.6)	26,261 (18.3)
Age (years)	Median (IQR)	71 (63-77)	71 (63-77)	72 (64-78)	70 (61-77)
	Mean (SD)	69.76 (10.77)	70.03 (10.1)	70.76 (10.06)	68.95 (11.38)
	Min, Max	35, 103	35, 100	35, 102	35, 103
Male	No	133,885 (45.8)	19,717 (45.5)	48,731 (46.3)	65,437 (45.6)
	Yes	158,212 (54.2)	23,591 (54.5)	56,518 (53.7)	78,103 (54.4)
Grouped BMI	Underweight (<18.5)	11,366 (3.9)	1,683 (3.9)	4,740 (4.5)	4,943 (3.4)
	Normal (18.5-24.9)	89,275 (30.6)	12,949 (29.9)	31,865 (30.3)	44,461 (31.0)
	Overweight (25-29.9)	95,543 (32.7)	14,019 (32.4)	33,514 (31.8)	48,010 (33.4)
	Obese I (30-34.9)	55,582 (19.0)	8,593 (19.8)	20,281 (19.3)	26,708 (18.6)
	Obese II (35-39.9)	22,082 (7.6)	3,572 (8.2)	8,386 (8.0)	10,124 (7.1)
	Obese III (40+)	10,018 (3.4)	1,581 (3.7)	3,948 (3.8)	4,489 (3.1)
	Missing	8,231 (2.8)	911 (2.1)	2,515 (2.4)	4,805 (3.3)
Smoking status	Never	0 (0.0)	0 (0.0)	0 (0.0)	0 (0.0)
	Former	184,571 (63.2)	26,040 (60.1)	69,740 (66.3)	88,791 (61.9)
	Current	107,526 (36.8)	17,268 (39.9)	35,509 (33.7)	54,749 (38.1)
	Missing	0 (0.0)	0 (0.0)	0 (0.0)	0 (0.0)

Ethnicity	White	219,008 (75.0)	32,498 (75.0)	79,735 (75.8)	106,775 (74.4)
	Mixed	676 (0.2)	92 (0.2)	182 (0.2)	402 (0.3)
	Asian or Asian British	3,369 (1.2)	260 (0.6)	836 (0.8)	2,273 (1.6)
	Black	1,210 (0.4)	103 (0.2)	301 (0.3)	806 (0.6)
	Other	1,061 (0.4)	112 (0.3)	289 (0.3)	660 (0.5)
	Unknown	66,773 (22.9)	10,243 (23.7)	23,906 (22.7)	32,624 (22.7)
Index of Multiple Deprivation (IMD)	Least Deprived	58,944 (20.2)	8,066 (18.6)	19,896 (18.9)	30,982 (21.6)
	2	58,848 (20.1)	8,426 (19.5)	20,629 (19.6)	29,793 (20.8)
	3	59,247 (20.3)	8,757 (20.2)	21,244 (20.2)	29,246 (20.4)
	4	57,682 (19.7)	8,425 (19.5)	21,641 (20.6)	27,616 (19.2)
	Most Deprived	57,376 (19.6)	9,634 (22.2)	21,839 (20.7)	25,903 (18.0)
	Missing	0 (0.0)	0 (0.0)	0 (0.0)	0 (0.0)
**Treatments**					
Single SABA	No	125,214 (42.9)	12,363 (28.5)	21,566 (20.5)	91,285 (63.6)
	Yes	166,883 (57.1)	30,945 (71.5)	83,683 (79.5)	52,255 (36.4)
High Dose ICS	No	266,397 (91.2)	43,308 (100.0)	79,782 (75.8)	143,307 (99.8)
	Yes	25,700 (8.8)	0 (0.0)	25,467 (24.2)	233 (0.2)
Low/Medium Dose ICS	No	204,825 (70.1)	43,308 (100.0)	22,743 (21.6)	138,774 (96.7)
	Yes	87,272 (29.9)	0 (0.0)	82,506 (78.4)	4,766 (3.3)
Single ICS	No	284,779 (97.5)	43,308 (100.0)	102,924 (97.8)	138,547 (96.5)
	Yes	7,318 (2.5)	0 (0.0)	2,325 (2.2)	4,993 (3.5)
Single SAMA	No	288,777 (98.9)	43,151 (99.6)	103,761 (98.6)	141,865 (98.8)
	Yes	3,320 (1.1)	157 (0.4)	1,488 (1.4)	1,675 (1.2)
Single LABA	No	284,018 (97.2)	40,875 (94.4)	104,394 (99.2)	138,749 (96.7)
	Yes	8,079 (2.8)	2,433 (5.6)	855 (0.8)	4,791 (3.3)

Single LAMA	No	204,395 (70.0)	38,718 (89.4)	59,766 (56.8)	105,911 (73.8)
	Yes	87,702 (30.0)	4,590 (10.6)	45,483 (43.2)	37,629 (26.2)
LABA ICS	No	216,545 (74.1)	43,308 (100.0)	29,697 (28.2)	143,540 (100.0)
	Yes	75,552 (25.9)	0 (0.0)	75,552 (71.8)	0 (0.0)
LABA LAMA	No	245,913 (84.2)	1,931 (4.5)	100,442 (95.4)	143,540 (100.0)
	Yes	46,184 (15.8)	41,377 (95.5)	4,807 (4.6)	0 (0.0)
LABA LAMA ICS	No	259,057 (88.7)	43,308 (100.0)	72,209 (68.6)	143,540 (100.0)
	Yes	33,040 (11.3)	0 (0.0)	33,040 (31.4)	0 (0.0)
Single LTRA	No	292,097 (100.0)	43,308 (100.0)	105,249 (100.0)	143,540 (100.0)
	Yes	0 (0.0)	0 (0.0)	0 (0.0)	0 (0.0)
**Clinical Conditions**					
Chronic kidney disease	No	242,158 (82.9)	35,570 (82.1)	86,886 (82.6)	119,702 (83.4)
	Yes	49,939 (17.1)	7,738 (17.9)	18,363 (17.4)	23,838 (16.6)
Diagnosed hypertension	No	147,010 (50.3)	21,607 (49.9)	50,954 (48.4)	74,449 (51.9)
	Yes	145,087 (49.7)	21,701 (50.1)	54,295 (51.6)	69,091 (48.1)
Heart Failure	No	267,454 (91.6)	39,445 (91.1)	95,283 (90.5)	132,726 (92.5)
	Yes	24,643 (8.4)	3,863 (8.9)	9,966 (9.5)	10,814 (7.5)
Other Heart Diseases	No	226,533 (77.6)	33,253 (76.8)	81,128 (77.1)	112,152 (78.1)
	Yes	65,564 (22.4)	10,055 (23.2)	24,121 (22.9)	31,388 (21.9)
Cancer	No	252,004 (86.3)	37,073 (85.6)	90,171 (85.7)	124,760 (86.9)
	Yes	40,093 (13.7)	6,235 (14.4)	15,078 (14.3)	18,780 (13.1)
Diabetes Severity	No Diabetes	221,389 (75.8)	32,913 (76.0)	79,549 (75.6)	108,927 (75.9)

	Diabetes, not severe	51,349 (17.6)	7,586 (17.5)	19,030 (18.1)	24,733 (17.2)
	Diabetes, severe	18,577 (6.4)	2,712 (6.3)	6,366 (6.0)	9,499 (6.6)
	Diabetes, no Hb1AC	782 (0.3)	97 (0.2)	304 (0.3)	381 (0.3)
Recent Statin	No	150,591 (51.6)	20,531 (47.4)	50,912 (48.4)	79,148 (55.1)
	Yes	141,506 (48.4)	22,777 (52.6)	54,337 (51.6)	64,392 (44.9)
Flu vaccine	No	73,937 (25.3)	8,689 (20.1)	19,917 (18.9)	45,331 (31.6)
	Yes	218,160 (74.7)	34,619 (79.9)	85,332 (81.1)	98,209 (68.4)
Pneumococcal Vaccine	No	231,561 (79.3)	32,285 (74.5)	83,943 (79.8)	115,333 (80.3)
	Yes	60,536 (20.7)	11,023 (25.5)	21,306 (20.2)	28,207 (19.7)
Exacerbation in last year	No	236,381 (80.9)	34,774 (80.3)	77,897 (74.0)	123,710 (86.2)
	Yes	55,716 (19.1)	8,534 (19.7)	27,352 (26.0)	19,830 (13.8)
Asthma ever	No	236,837 (81.1)	37,731 (87.1)	76,063 (72.3)	123,043 (85.7)
	Yes	55,260 (18.9)	5,577 (12.9)	29,186 (27.7)	20,497 (14.3)
Immunosuppressed (combination algorithm)	No	291,348 (99.7)	43,211 (99.8)	105,013 (99.8)	143,124 (99.7)
	Yes	749 (0.3)	97 (0.2)	236 (0.2)	416 (0.3)
GP consultation count	Median (IQR)	9 (5-16)	10 (6-16)	10 (6-17)	8 (4-14)
	Mean (SD)	12.21 (11.72)	12.73 (11.36)	13.46 (12.34)	11.15 (11.25)
	Min, Max	0, 510	0, 276	0, 306	0, 510
Exacerbation Count	Median (IQR)	0 (0-0)	0 (0-0)	0 (0-1)	0 (0-0)
	Mean (SD)	.26 (.62)	.26 (.6)	.38 (.77)	.17 (.48)
	Min, Max	0, 12	0, 9	0, 12	0, 8

Table 2. Demographic and Clinical Characteristics - Asthma

		Total	SABA only	ICS (Low/Medium Dose)	ICS (High Dose)	Other
**Total Included**		**1,285,855 (100.0)**	**108,441 (100.0)**	**608,972 (100.0)**	**101,077 (100.0)**	**467,365 (100.0)**
**Demographics**						
Grouped age	18 - <40	417,459 (32.5)	36,264 (33.4)	144,955 (23.8)	18,836 (18.6)	217,404 (46.5)
	40 - <50	235,490 (18.3)	22,067 (20.3)	107,835 (17.7)	18,459 (18.3)	87,129 (18.6)
	50 - < 60	253,288 (19.7)	21,852 (20.2)	130,434 (21.4)	23,752 (23.5)	77,250 (16.5)
	60 - <70	181,994 (14.2)	13,974 (12.9)	105,897 (17.4)	18,970 (18.8)	43,153 (9.2)
	70 - <80	130,684 (10.2)	9,209 (8.5)	79,810 (13.1)	13,904 (13.8)	27,761 (5.9)
	80+	66,940 (5.2)	5,075 (4.7)	40,041 (6.6)	7,156 (7.1)	14,668 (3.1)
Age (years)	Median (IQR)	49 (35-62)	48 (35-60)	53 (40-66)	55 (44-67)	41 (29-55)
	Mean (SD)	49.33 (17.9)	48.31 (17.38)	53.13 (17.37)	55.01 (16.35)	43.37 (17.29)
	Min, Max	18, 106	18, 106	18, 106	18, 106	18, 105
Male	No	750,523 (58.4)	61,827 (57.0)	363,526 (59.7)	62,487 (61.8)	262,683 (56.2)
	Yes	535,332 (41.6)	46,614 (43.0)	245,446 (40.3)	38,590 (38.2)	204,682 (43.8)
Grouped BMI	Underweight (<18.5)	19,140 (1.5)	1,637 (1.5)	7,623 (1.3)	1,147 (1.1)	8,733 (1.9)
	Normal (18.5-24.9)	340,745 (26.5)	28,141 (26.0)	153,022 (25.1)	20,955 (20.7)	138,627 (29.7)
	Overweight (25-29.9)	394,872 (30.7)	32,874 (30.3)	196,599 (32.3)	30,763 (30.4)	134,636 (28.8)
	Obese I (30-34.9)	239,387 (18.6)	20,069 (18.5)	122,212 (20.1)	22,297 (22.1)	74,809 (16.0)
	Obese II (35-39.9)	112,084 (8.7)	9,497 (8.8)	56,837 (9.3)	11,891 (11.8)	33,859 (7.2)
	Obese III (40+)	67,819 (5.3)	5,937 (5.5)	34,102 (5.6)	8,248 (8.2)	19,532 (4.2)
	Missing	111,808 (8.7)	10,286 (9.5)	38,577 (6.3)	5,776 (5.7)	57,169 (12.2)
Smoking status	Never	590,091 (45.9)	45,384 (41.9)	268,922 (44.2)	41,231 (40.8)	234,554 (50.2)
	Former	498,181 (38.7)	42,272 (39.0)	254,334 (41.8)	44,137 (43.7)	157,438 (33.7)
	Current	195,461 (15.2)	20,625 (19.0)	85,414 (14.0)	15,665 (15.5)	73,757 (15.8)
	Missing	2,122 (0.2)	160 (0.1)	302 (0.0)	44 (0.0)	1,616 (0.3)

Ethnicity	White	883,635 (68.7)	74,402 (68.6)	428,142 (70.3)	71,303 (70.5)	309,788 (66.3)
	Mixed	12,941 (1.0)	978 (0.9)	5,028 (0.8)	838 (0.8)	6,097 (1.3)
	Asian or Asian British	72,109 (5.6)	5,698 (5.3)	32,357 (5.3)	5,866 (5.8)	28,188 (6.0)
	Black	20,499 (1.6)	1,546 (1.4)	8,139 (1.3)	1,455 (1.4)	9,359 (2.0)
	Other	12,202 (0.9)	892 (0.8)	4,863 (0.8)	881 (0.9)	5,566 (1.2)
	Unknown	284,469 (22.1)	24,925 (23.0)	130,443 (21.4)	20,734 (20.5)	108,367 (23.2)
Index of Multiple Deprivation (IMD)	Least Deprived	260,479 (20.3)	21,087 (19.4)	124,406 (20.4)	17,776 (17.6)	97,210 (20.8)
	2	261,702 (20.4)	21,705 (20.0)	124,312 (20.4)	18,956 (18.8)	96,729 (20.7)
	3	258,640 (20.1)	22,016 (20.3)	121,926 (20.0)	20,217 (20.0)	94,481 (20.2)
	4	258,487 (20.1)	22,392 (20.6)	121,117 (19.9)	21,478 (21.2)	93,500 (20.0)
	Most Deprived	246,547 (19.2)	21,241 (19.6)	117,211 (19.2)	22,650 (22.4)	85,445 (18.3)
	Missing	0 (0.0)	0 (0.0)	0 (0.0)	0 (0.0)	0 (0.0)
**Treatments**						
Single SABA	No	679,705 (52.9)	0 (0.0)	191,964 (31.5)	22,855 (22.6)	464,886 (99.5)
	Yes	606,150 (47.1)	108,441 (100.0)	417,008 (68.5)	78,222 (77.4)	2,479 (0.5)
High Dose ICS	No	1181864 (91.9)	108,441 (100.0)	606,058 (99.5)	0 (0.0)	467,365 (100.0)
	Yes	103,991 (8.1)	0 (0.0)	2,914 (0.5)	101,077 (100.0)	0 (0.0)
Low/Medium Dose ICS	No	669,336 (52.1)	108,441 (100.0)	0 (0.0)	93,530 (92.5)	467,365 (100.0)
	Yes	616,519 (47.9)	0 (0.0)	608,972 (100.0)	7,547 (7.5)	0 (0.0)
Single ICS	No	994,887 (77.4)	108,441 (100.0)	328,092 (53.9)	90,989 (90.0)	467,365 (100.0)
	Yes	290,968 (22.6)	0 (0.0)	280,880 (46.1)	10,088 (10.0)	0 (0.0)
Single SAMA	No	1283033 (99.8)	108,325 (99.9)	607,342 (99.7)	100,181 (99.1)	467,185 (100.0)
	Yes	2,822 (0.2)	116 (0.1)	1,630 (0.3)	896 (0.9)	180 (0.0)

Single LABA	No	1277335 (99.3)	107,898 (99.5)	603,012 (99.0)	99,574 (98.5)	466,851 (99.9)
	Yes	8,520 (0.7)	543 (0.5)	5,960 (1.0)	1,503 (1.5)	514 (0.1)
Single LAMA	No	1269483 (98.7)	108,441 (100.0)	600,935 (98.7)	92,742 (91.8)	467,365 (100.0)
	Yes	16,372 (1.3)	0 (0.0)	8,037 (1.3)	8,335 (8.2)	0 (0.0)
LABA ICS	No	854,214 (66.4)	108,441 (100.0)	270,790 (44.5)	7,618 (7.5)	467,365 (100.0)
	Yes	431,641 (33.6)	0 (0.0)	338,182 (55.5)	93,459 (92.5)	0 (0.0)
LABA LAMA	No	1285498 (100.0)	108,441 (100.0)	608,736 (100.0)	100,956 (99.9)	467,365 (100.0)
	Yes	357 (0.0)	0 (0.0)	236 (0.0)	121 (0.1)	0 (0.0)
LABA LAMA ICS	No	1284592 (99.9)	108,441 (100.0)	607,795 (99.8)	100,991 (99.9)	467,365 (100.0)
	Yes	1,263 (0.1)	0 (0.0)	1,177 (0.2)	86 (0.1)	0 (0.0)
Single LTRA	No	1215996 (94.6)	108,441 (100.0)	568,504 (93.4)	78,023 (77.2)	461,028 (98.6)
	Yes	69,859 (5.4)	0 (0.0)	40,468 (6.6)	23,054 (22.8)	6,337 (1.4)
**Clinical Conditions**						
Chronic kidney disease	No	1224572 (95.2)	103,341 (95.3)	573,525 (94.2)	94,026 (93.0)	453,680 (97.1)
	Yes	61,283 (4.8)	5,100 (4.7)	35,447 (5.8)	7,051 (7.0)	13,685 (2.9)
Diagnosed hypertension	No	977,445 (76.0)	82,598 (76.2)	432,452 (71.0)	67,206 (66.5)	395,189 (84.6)
	Yes	308,410 (24.0)	25,843 (23.8)	176,520 (29.0)	33,871 (33.5)	72,176 (15.4)
Heart Failure	No	1263403 (98.3)	106,459 (98.2)	596,359 (97.9)	98,081 (97.0)	462,504 (99.0)
	Yes	22,452 (1.7)	1,982 (1.8)	12,613 (2.1)	2,996 (3.0)	4,861 (1.0)
Other Heart Diseases	No	1206759 (93.8)	101,549 (93.6)	565,066 (92.8)	91,929 (90.9)	448,215 (95.9)
	Yes	79,096 (6.2)	6,892 (6.4)	43,906 (7.2)	9,148 (9.1)	19,150 (4.1)

Cancer	No	1218429 (94.8)	102,740 (94.7)	571,390 (93.8)	94,325 (93.3)	449,974 (96.3)
	Yes	67,426 (5.2)	5,701 (5.3)	37,582 (6.2)	6,752 (6.7)	17,391 (3.7)
Diabetes Severity						
	No Diabetes	1126834 (87.6)	93,589 (86.3)	523,874 (86.0)	83,064 (82.2)	426,307 (91.2)
	Diabetes, not severe	105,907 (8.2)	8,946 (8.2)	57,866 (9.5)	12,254 (12.1)	26,841 (5.7)
	Diabetes, severe	48,890 (3.8)	5,551 (5.1)	25,283 (4.2)	5,436 (5.4)	12,620 (2.7)
	Diabetes, no Hb1AC	4,224 (0.3)	355 (0.3)	1,949 (0.3)	323 (0.3)	1,597 (0.3)
Recent Statin	No	1064727 (82.8)	90,340 (83.3)	475,969 (78.2)	74,854 (74.1)	423,564 (90.6)
	Yes	221,128 (17.2)	18,101 (16.7)	133,003 (21.8)	26,223 (25.9)	43,801 (9.4)
Flu vaccine	No	667,467 (51.9)	65,191 (60.1)	240,861 (39.6)	36,016 (35.6)	325,399 (69.6)
	Yes	618,388 (48.1)	43,250 (39.9)	368,111 (60.4)	65,061 (64.4)	141,966 (30.4)
Pneumococcal Vaccine	No	1196221 (93.0)	101,846 (93.9)	557,804 (91.6)	91,105 (90.1)	445,466 (95.3)
	Yes	89,634 (7.0)	6,595 (6.1)	51,168 (8.4)	9,972 (9.9)	21,899 (4.7)
Exacerbation in last year	No	1076578 (83.7)	93,231 (86.0)	487,282 (80.0)	64,351 (63.7)	431,714 (92.4)
	Yes	209,277 (16.3)	15,210 (14.0)	121,690 (20.0)	36,726 (36.3)	35,651 (7.6)
Immunosuppressed (combination algorithm)	No	1281979 (99.7)	108,056 (99.6)	607,221 (99.7)	100,762 (99.7)	465,940 (99.7)
	Yes	3,876 (0.3)	385 (0.4)	1,751 (0.3)	315 (0.3)	1,425 (0.3)
GP consultation count	Median (IQR)	6 (3-12)	6 (3-12)	7 (4-13)	9 (5-16)	5 (2-10)
	Mean (SD)	9.3 (10.87)	9.26 (10.65)	10.25 (11.2)	12.8 (13.48)	7.31 (9.39)
	Min, Max	0, 604	0, 296	0, 548	0, 604	0, 604
Exacerbation Count	Median (IQR)	0 (0-0)	0 (0-0)	0 (0-0)	0 (0-1)	0 (0-0)
	Mean (SD)	.3 (1.03)	.24 (.95)	.35 (1.09)	.81 (1.71)	.13 (.68)
	Min, Max	0, 17	0, 16	0, 17	0, 17	0, 16

Table 3. Association between current ICS use and COVID-19 Death* in ONS – All COPD analyses

					Univariable		Age/Sex Adjusted		Age/Sex and Comorbidity Adjusted	
		Events	Person-weeks	Rate per 1,000	HR	95% CI	HR	95% CI	HR	95% CI
Main Model	LABA/LAMA	91	406580	0.22	1.00 (ref)		1.00 (ref)		1.00 (ref)	
	ICS	338	986485	0.34	1.53	1.22 - 1.93	1.43	1.13 - 1.80	1.39	1.10 - 1.76
Negative Control (Non-COVID Death)	LABA/LAMA	318	406580	0.78	1.00 (ref)		1.00 (ref)		1.00 (ref)	
	ICS	1021	986485	1.03	1.32	1.17 - 1.50	1.26	1.11 - 1.43	1.23	1.08 - 1.40
Sensitivity: Ethnicity	LABA/LAMA	77	305092	0.25	1.00 (ref)		1.00 (ref)		1.00 (ref)	
	ICS	242	747458	0.32	1.28	0.99 - 1.66	1.19	0.92 - 1.54	1.14	0.87 - 1.48
Sensitivity: Exposure	LABA/LAMA	91	406580	0.22	1.00 (ref)		1.00 (ref)		1.00 (ref)	
	ICS Dual	89	280458	0.32	1.42	1.06 - 1.90	1.27	0.95 - 1.70	1.29	0.96 - 1.74
	ICS Triple	249	706027	0.35	1.58	1.24 - 2.00	1.49	1.17 - 1.90	1.43	1.12 - 1.83
Sensitivity: LAMA mono	LABA/LAMA	163	747092	0.22	1.00 (ref)		1.00 (ref)		1.00 (ref)	
	ICS	338	986485	0.34	1.57	1.30 - 1.89	1.48	1.23 - 1.79	1.41	1.17 - 1.70

*Unless otherwise specified

Table 4. Current ICS use and COVID-19 Death in ONS, Age Interaction - COPD Population

					Univariable			Age/Sex Adjusted			Age/Sex and Comorbidity Adjusted		
		Events	Person-weeks	Rate per 1,000	HR	95% CI	p (inter)	HR	95% CI	p (inter)	HR	95% CI	p (inter)
**Age**							0.96			0.97			0.97
<60													
	LABA/LAMA	[Redacted]	64919	0.03	1.00 (ref)			1.00 (ref)			1.00 (ref)		
	ICS	[Redacted]	138559	0.05	1.64	0.34 - 7.89		1.67	0.35 - 8.02		1.53	0.32 - 7.35	
60 - <70													
	LABA/LAMA	7	118589	0.06	1.00 (ref)			1.00 (ref)			1.00 (ref)		
	ICS	22	277653	0.08	1.34	0.57 - 3.14		1.35	0.58 - 3.17		1.24	0.53 - 2.90	
70 - <80													
	LABA/LAMA	31	151217	0.21	1.00 (ref)			1.00 (ref)			1.00 (ref)		
	ICS	121	378488	0.32	1.56	1.05 - 2.31		1.56	1.05 - 2.31		1.49	1.00 - 2.22	
80+													
	LABA/LAMA	51	71855	0.71	1.00 (ref)			1.00 (ref)			1.00 (ref)		
	ICS	188	191785	0.98	1.38	1.01 - 1.88		1.39	1.02 - 1.89		1.36	1.00 - 1.87	

Table 5. Testing the PH assumption for COVID-19 Death in ONS- COPD Population

	Univariable	Age/Sex Adjusted	Age/Sex and Comorbidity Adjusted
	p-value	p-value	p-value
Treatment Exposure	0.083	0.069	0.108

Figure 4. Schoenfeld residuals, unadjusted

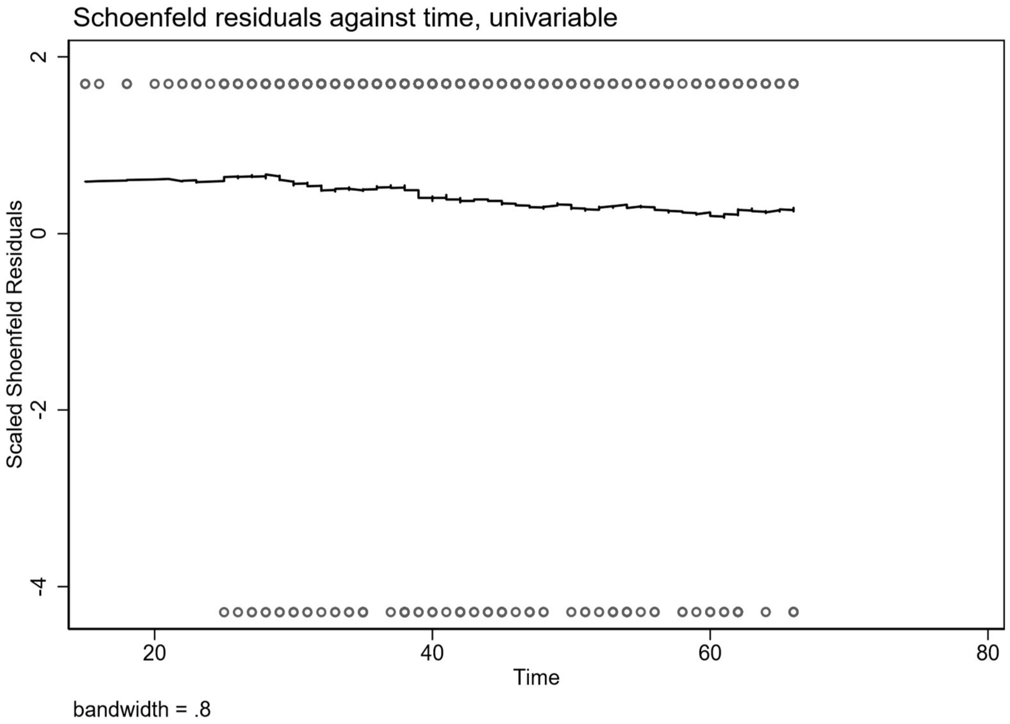

Figure 5. Schoenfeld residuals, age/sex adjusted

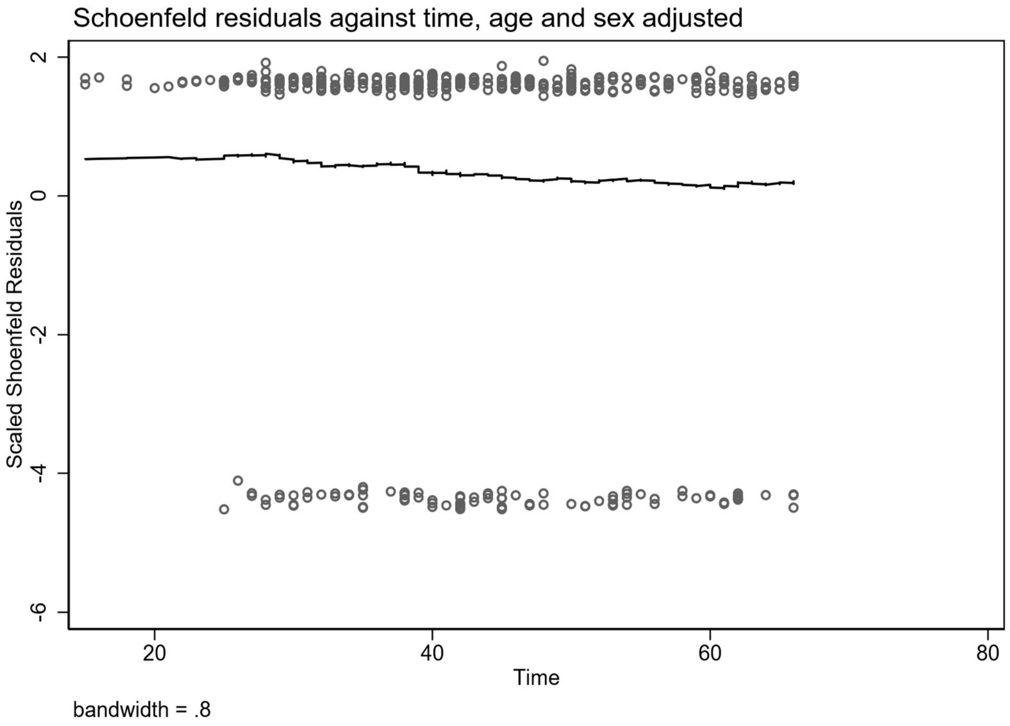

Figure 6. Schoenfeld residuals, fully adjusted

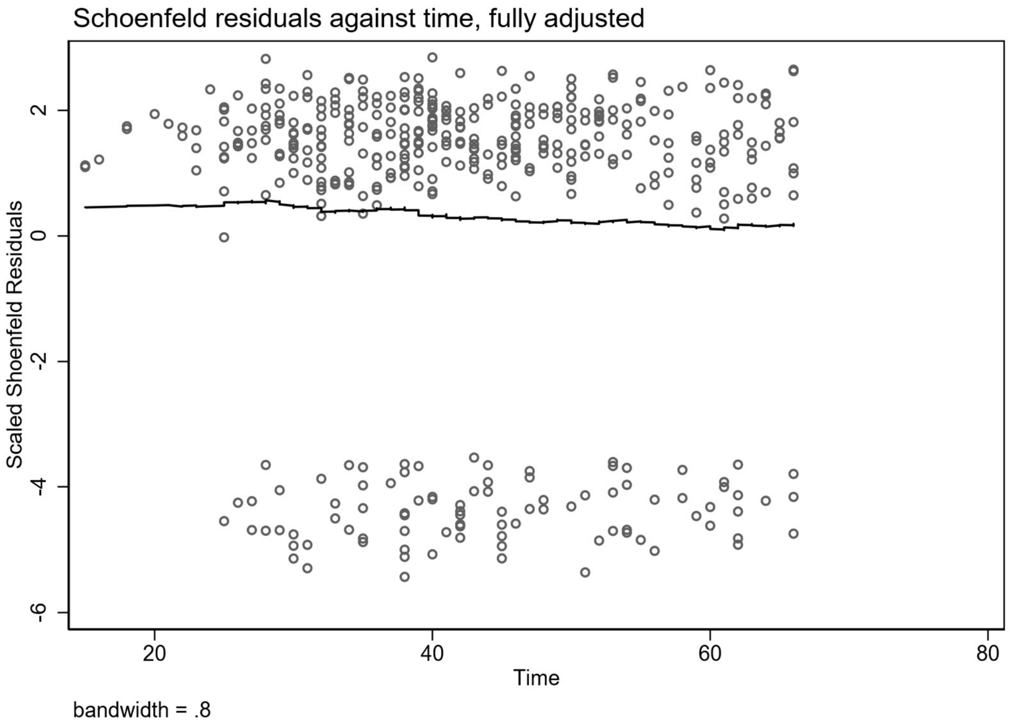

Table 6. 1 by 1 comorbidity adjustments (after age/sex and strata adjustments) - COPD population

	HR	95% CI
Evidence of obesity (4 categories)		
LABA/LAMA Combination	1.00 (ref)	
ICS Combination	1.4	1.11 - 1.77
Smoking status (missing set to non)		
LABA/LAMA Combination	1.00 (ref)	
ICS Combination	1.37	1.08 - 1.73
Index of Multiple Deprivation (IMD)		
LABA/LAMA Combination	1.00 (ref)	
ICS Combination	1.38	1.10 - 1.75
Chronic kidney disease		
LABA/LAMA Combination	1.00 (ref)	
ICS Combination	1.42	1.13 - 1.80
Diagnosed hypertension		
LABA/LAMA Combination	1.00 (ref)	
ICS Combination	1.39	1.10 - 1.76
Heart Failure		
LABA/LAMA Combination	1.00 (ref)	
ICS Combination	1.4	1.10 - 1.77
Other Heart Diseases		
LABA/LAMA Combination	1.00 (ref)	
ICS Combination	1.4	1.11 - 1.77
Diabetes Severity		
LABA/LAMA Combination	1.00 (ref)	
ICS Combination	1.4	1.10 - 1.76
Cancer		
LABA/LAMA Combination	1.00 (ref)	
ICS Combination	1.39	1.10 - 1.76
Recent Statin		
LABA/LAMA Combination	1.00 (ref)	
ICS Combination	1.39	1.10 - 1.76
Flu vaccine		
LABA/LAMA Combination	1.00 (ref)	
ICS Combination	1.39	1.10 - 1.76
Pneumococcal Vaccine		
LABA/LAMA Combination	1.00 (ref)	
ICS Combination	1.39	1.10 - 1.76

Exacerbation in last year		
LABA/LAMA Combination	1.00 (ref)	
ICS Combination	1.35	1.07 - 1.71
Asthma ever		
LABA/LAMA Combination	1.00 (ref)	
ICS Combination	1.43	1.13 - 1.81
Immunosuppressed (combination algorithm)		
LABA/LAMA Combination	1.00 (ref)	
ICS Combination	1.39	1.10 - 1.76

Table 7. Additional adjustments - COPD

**Adjustment Set**		**HR**	**95% CI**
Main Model	LABA/LAMA Combination	1.00 (ref)	
	ICS Combination	1.39	1.10 - 1.76
Wo. Exposure History	LABA/LAMA Combination	1.00 (ref)	
	ICS Combination	1.43	1.13 - 1.81
W oral steroids	LABA/LAMA Combination	1.00 (ref)	
	ICS Combination	1.38	1.09 - 1.75

Table 8. Association between current ICS use and COVID-19 Death* in ONS – All Asthma Analyses

					Univariable		Age/Sex Adjusted		Age/Sex and Comorbidity Adjusted	
		N	Person-weeks	Rate per 1,000	HR	95% CI	HR	95% CI	HR	95% CI
Main Model	SABA only	49	1021455	0.05	1.00 (ref)		1.00 (ref)		1.00 (ref)	
	ICS (Low/Medium Dose)	375	5736155	0.07	1.36	1.01 - 1.84	1.02	0.76 - 1.37	1.14	0.85 - 1.54
	ICS (High Dose)	105	951654	0.11	2.3	1.64 - 3.23	1.61	1.15 - 2.27	1.55	1.10 - 2.18
Negative Control (Non-COVID Death)	SABA only	168	1021455	0.16	1.00 (ref)		1.00 (ref)	1.00 (ref)		
	ICS (Low/Medium Dose)	934	5736155	0.16	0.99	0.84 - 1.17	0.75	0.63 - 0.88	0.83	0.70 - 0.98
	ICS (High Dose)	206	951654	0.22	1.32	1.07 - 1.61	0.93	0.76 - 1.14	0.94	0.76 - 1.15
Sensitivity: Ethnicity	SABA only	18	700867	0.03	1.00 (ref)		1.00 (ref)		1.00 (ref)	
	ICS (Low/Medium Dose)	208	4033235	0.05	2.01	1.24 - 3.25	1.5	0.93 - 2.44	1.65	1.02 - 2.68
	ICS (High Dose)	63	671400	0.09	3.65	2.16 - 6.17	2.62	1.55 - 4.42	2.5	1.48 - 4.24
Sensitivity: Population Definition	SABA only	63	1262743	0.05	1.00 (ref)		1.00 (ref)		1.00 (ref)	
	ICS (Low/Medium Dose)	398	5902652	0.07	1.35	1.04 - 1.76	1.03	0.79 - 1.34	1.16	0.89 - 1.51
	ICS (High Dose)	110	967728	0.11	2.28	1.67 - 3.11	1.62	1.19 - 2.21	1.55	1.13 - 2.12

* Unless otherwise specified

Table 9. Current ICS use and COVID-19 Death in ONS, Age Interaction - Asthma Population

					Univariable			Age/Sex Adjusted			Age/Sex and Comorbidity Adjusted		
		Events	Person-weeks	Rate per 1,000	HR	95% CI	p (inter)	HR	95% CI	p (inter)	HR	95% CI	p (inter)
**Age**							0.17			0.16			0.23
**<60**													
	SABA only	7	755817	0.01	1.00 (ref)			1.00 (ref)			1.00 (ref)		
	ICS (Low/Medium Dose)	49	3612609	0.01	1.46	0.66 - 3.23		1.48	0.67 - 3.26		1.5	0.68 - 3.32	
	ICS (High Dose)	14	575418	0.02	2.63	1.06 - 6.51		2.68	1.08 - 6.65		2.35	0.95 - 5.85	
**60 - <70**													
	SABA only	9	131582	0.07	1.00 (ref)			1.00 (ref)			1.00 (ref)		
	ICS (Low/Medium Dose)	42	997801	0.04	0.62	0.30 - 1.26		0.62	0.30 - 1.28		0.7	0.34 - 1.44	
	ICS (High Dose)	19	178673	0.11	1.55	0.70 - 3.44		1.58	0.71 - 3.49		1.46	0.66 - 3.24	
**70 - <80**													
	SABA only	14	86617	0.16	1.00 (ref)			1.00 (ref)			1.00 (ref)		
	ICS (Low/Medium Dose)	100	751199	0.13	0.82	0.47 - 1.44		0.82	0.47 - 1.44		0.93	0.53 - 1.63	
	ICS (High Dose)	33	130769	0.25	1.56	0.84 - 2.92		1.56	0.84 - 2.92		1.47	0.79 - 2.75	
**80+**													
	SABA only	19	47439	0.4	1.00 (ref)			1.00 (ref)			1.00 (ref)		
	ICS (Low/Medium Dose)	184	374546	0.49	1.23	0.76 - 1.97		1.22	0.76 - 1.95		1.41	0.88 - 2.27	
	ICS (High Dose)	39	66794	0.58	1.46	0.84 - 2.52		1.45	0.84 - 2.50		1.46	0.84 - 2.53	

Table 10. Testing the PH assumption for COVID-19 Death in ONS - Asthma Population

	Univariable	Age/Sex Adjusted	Age/Sex and Comorbidity Adjusted
	p-value	p-value	p-value
ICS (Low/Medium Dose)	0.489	0.461	0.577
ICS (High Dose)	0.688	0.668	0.644

Figure 7. Schoenfeld residuals, unadjusted ICS (low/medium-dose)

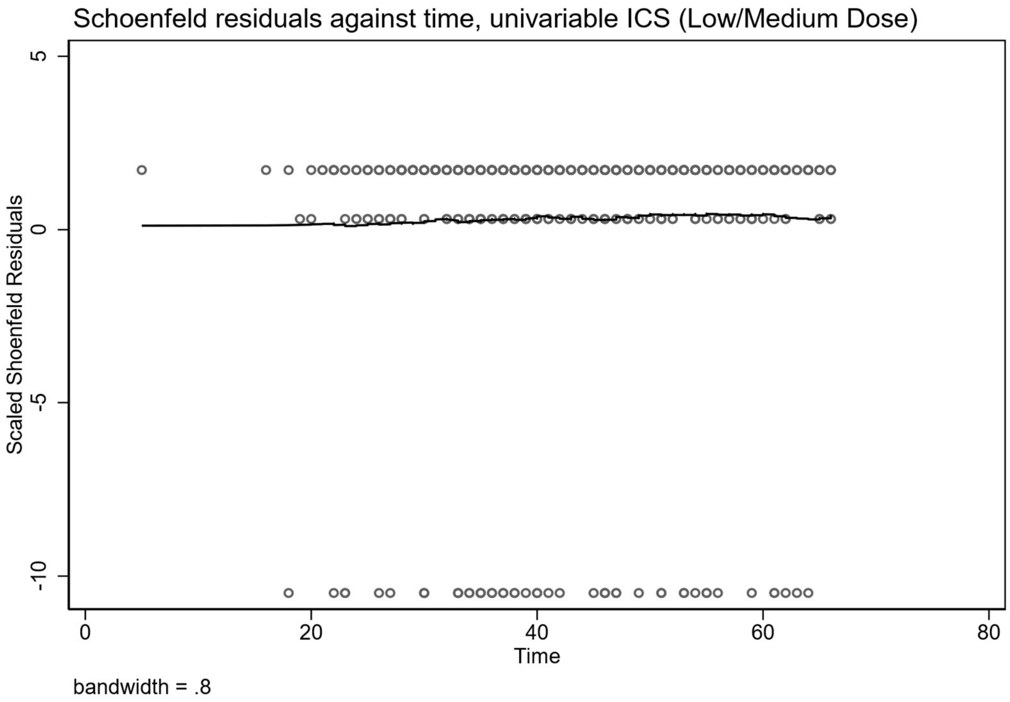

Figure 8. Schoenfeld residuals, age/sex adjusted ICS (low/medium dose)

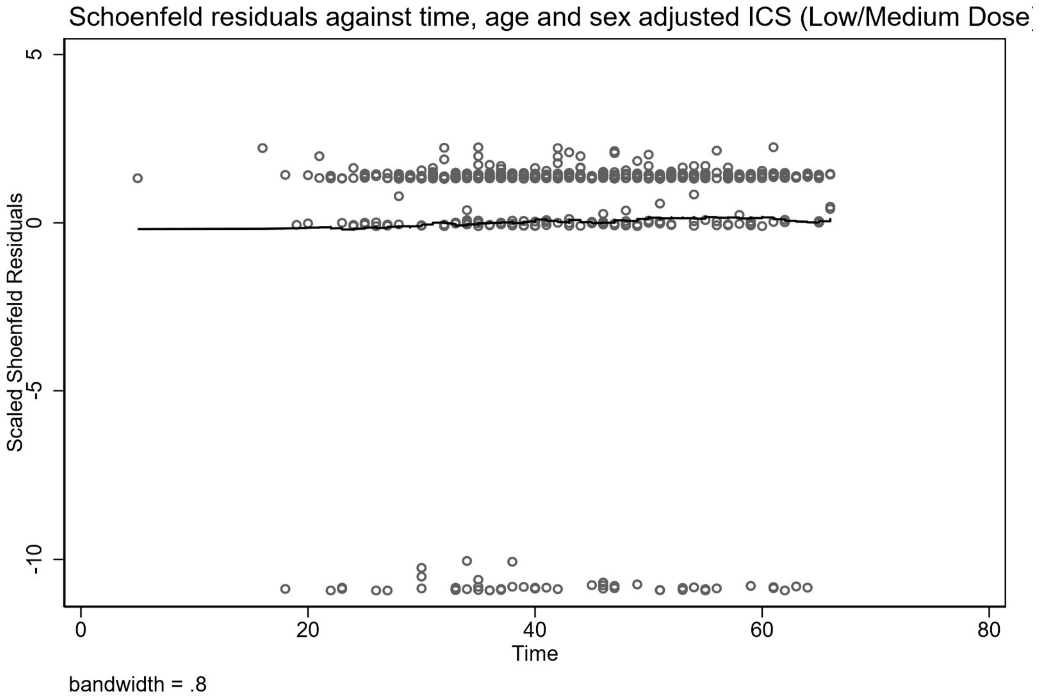

Figure 9. Schoenfeld residuals, fully adjusted ICS (low/medium dose)

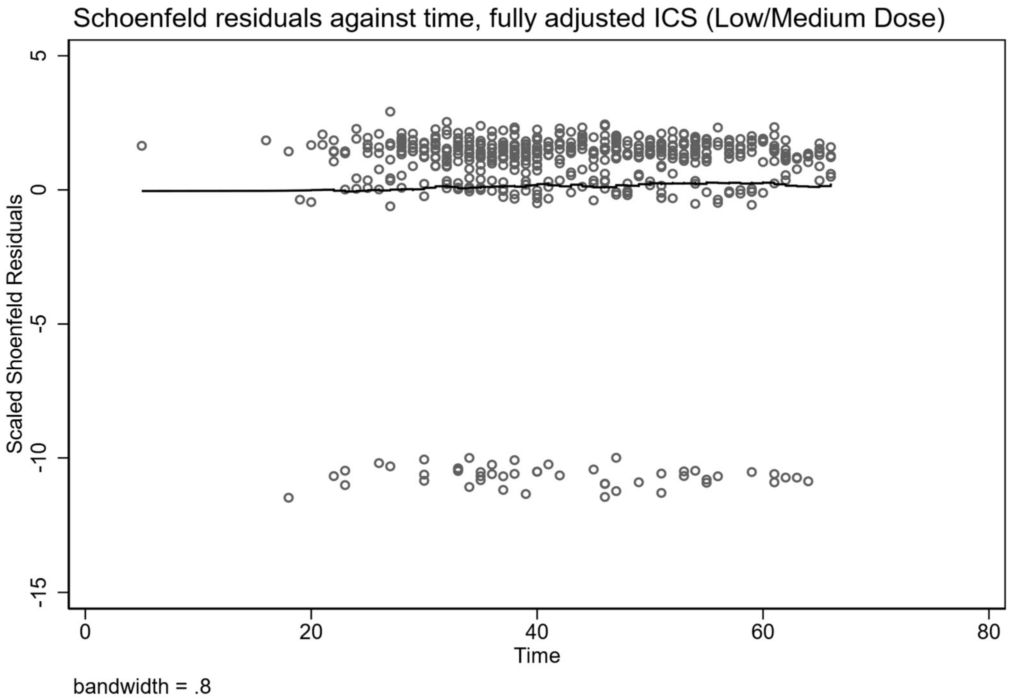

Figure 10. Schoenfeld residuals, unadjusted ICS (high-dose)

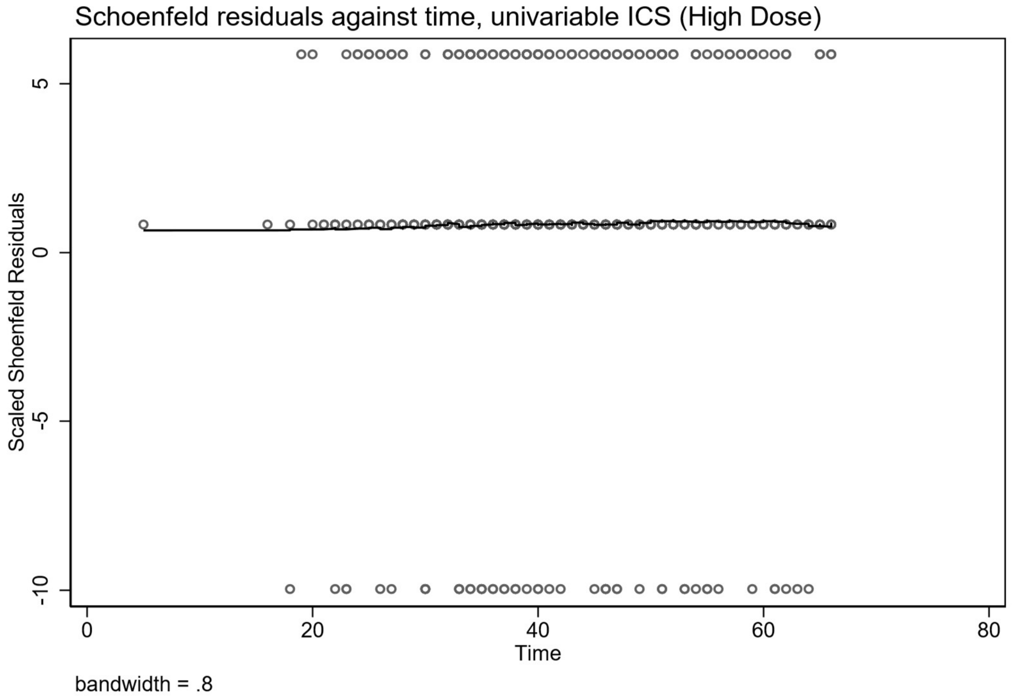

Figure 11. Schoenfeld residuals, age/sex adjusted ICS (high-dose)

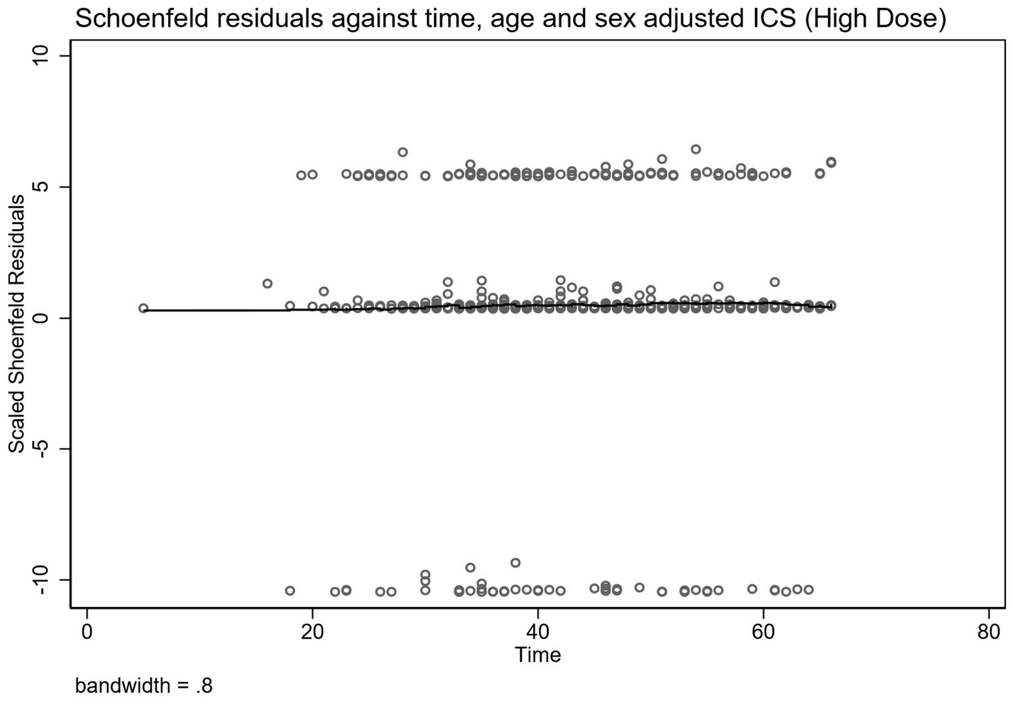

Figure 12. Schoenfeld residuals, fully adjusted ICS (high-dose)

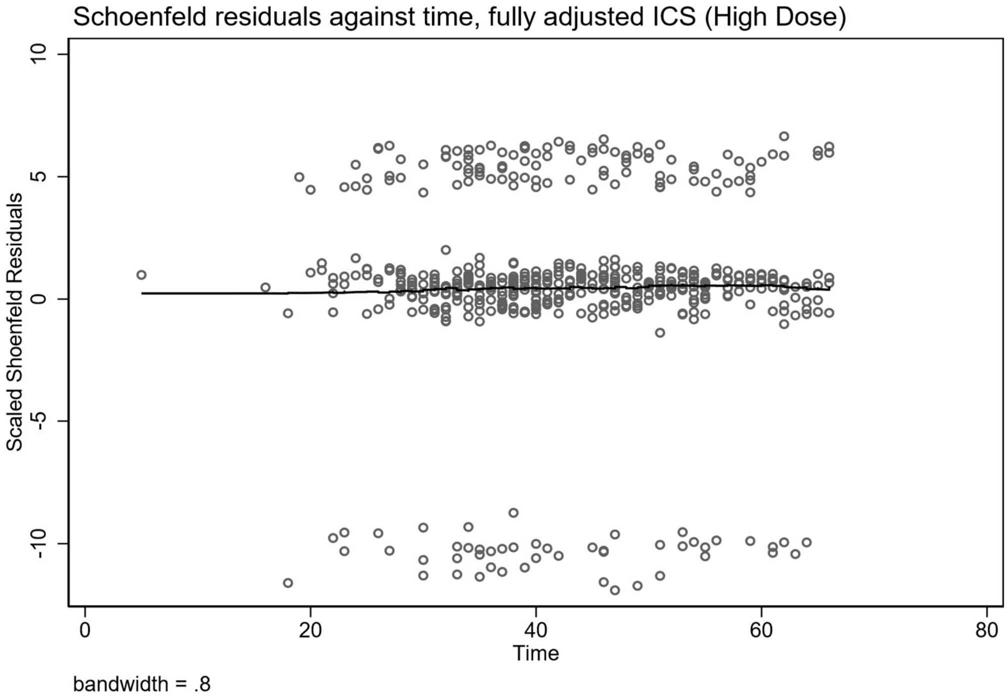

Table 11. 1 by 1 comorbidity adjustments (after age/sex and strata adjustments) - Asthma population

	HR	95% CI
Evidence of obesity (4 categories)		
SABA only	1.00 (ref)	
ICS (Low/Medium Dose)	1.07	0.80 - 1.45
ICS (High Dose)	1.55	1.10 - 2.17
Smoking status (missing set to non)		
SABA only	1.00 (ref)	
ICS (Low/Medium Dose)	1.06	0.79 - 1.43
ICS (High Dose)	1.59	1.13 - 2.23
Index of Multiple Deprivation (IMD)		
SABA only	1.00 (ref)	
ICS (Low/Medium Dose)	1.07	0.80 - 1.44
ICS (High Dose)	1.58	1.13 - 2.22
Chronic kidney disease		
SABA only	1.00 (ref)	
ICS (Low/Medium Dose)	1.08	0.80 - 1.46
ICS (High Dose)	1.61	1.14 - 2.26
Diagnosed hypertension		
SABA only	1.00 (ref)	
ICS (Low/Medium Dose)	1.07	0.79 - 1.44
ICS (High Dose)	1.58	1.13 - 2.23
Heart Failure		
SABA only	1.00 (ref)	
ICS (Low/Medium Dose)	1.08	0.80 - 1.45
ICS (High Dose)	1.59	1.13 - 2.23
Other Heart Diseases		
SABA only	1.00 (ref)	
ICS (Low/Medium Dose)	1.08	0.80 - 1.45
ICS (High Dose)	1.6	1.14 - 2.24
Diabetes Severity		
SABA only	1.00 (ref)	
ICS (Low/Medium Dose)	1.11	0.82 - 1.50
ICS (High Dose)	1.61	1.15 - 2.27
Cancer		
SABA only	1.00 (ref)	
ICS (Low/Medium Dose)	1.07	0.79 - 1.44
ICS (High Dose)	1.59	1.13 - 2.23
Recent Statin		
SABA only	1.00 (ref)	

ICS (Low/Medium Dose)	1.06	0.79 - 1.43
ICS (High Dose)	1.58	1.12 - 2.22
Flu vaccine		
SABA only	1.00 (ref)	
ICS (Low/Medium Dose)	1.08	0.80 - 1.45
ICS (High Dose)	1.61	1.15 - 2.27
Pneumococcal Vaccine		
SABA only	1.00 (ref)	
ICS (Low/Medium Dose)	1.07	0.79 - 1.44
ICS (High Dose)	1.59	1.13 - 2.24
Exacerbation in last year		
SABA only	1.00 (ref)	
ICS (Low/Medium Dose)	1.05	0.78 - 1.41
ICS (High Dose)	1.5	1.06 - 2.11
Immunosuppressed (combination algorithm)		
SABA only	1.00 (ref)	
ICS (Low/Medium Dose)	1.07	0.79 - 1.44
ICS (High Dose)	1.59	1.13 - 2.24

Table 12. Additional adjustments - asthma

Adjustment Set		HR	95% CI
**Main Model**	SABA only	1.00 (ref)	
	ICS (Low/Medium Dose)	1.14	0.85 - 1.54
	ICS (High Dose)	1.55	1.10 - 2.18
**Wo. Exposure History**	SABA only	1.00 (ref)	
	ICS (Low/Medium Dose)	1.16	0.86 - 1.56
	ICS (High Dose)	1.62	1.15 - 2.28
**W oral steroids**	SABA only	1.00 (ref)	
	ICS (Low/Medium Dose)	1.16	0.86 - 1.56
	ICS (High Dose)	1.6	1.13 - 2.25

Figure 13. E-value for the lower 95% CI and point estimate for the ICS COPD association, assuming the true effect is 1.0

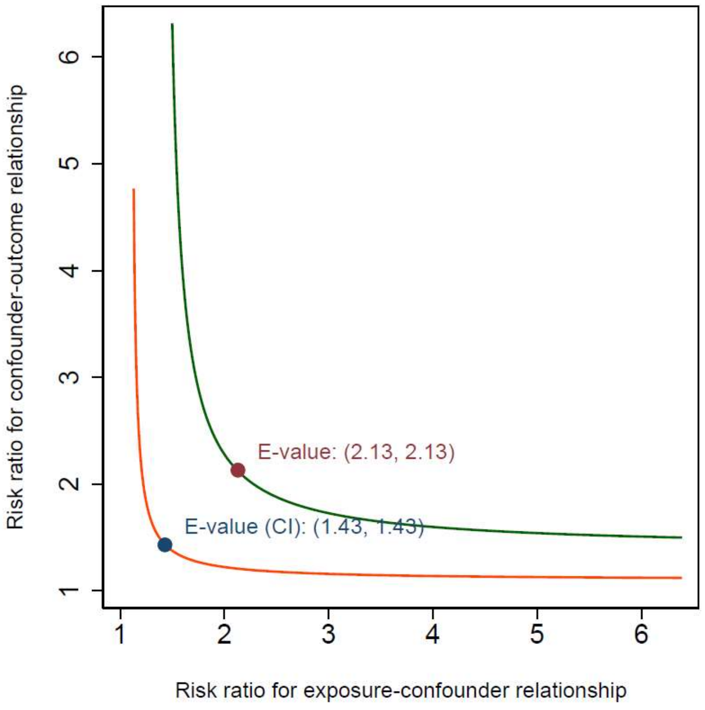

Figure 14. E-value for the lower 95% CI and point estimate for the high-dose ICS asthma association, assuming the true effect is 0.8

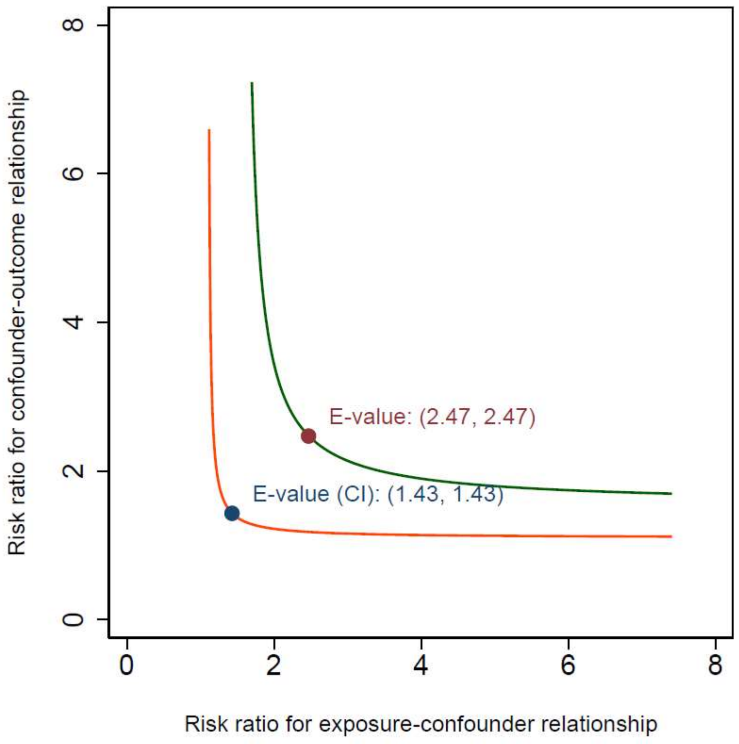

Figure 15. E-value for the lower 95% CI and point estimate for the ICS COPD association, assuming the true effect is 0.8

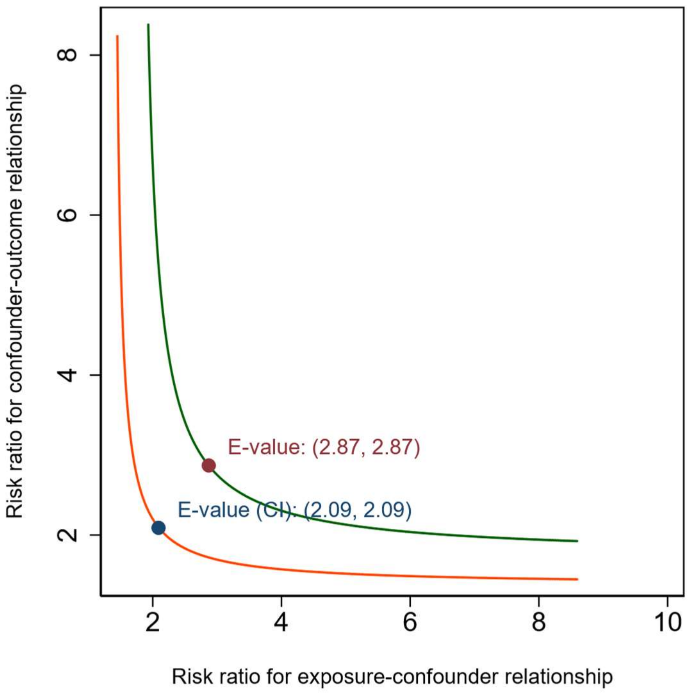

Figure 16. E-value for the lower 95% CI and point estimate for the high-dose ICS asthma association, assuming the true effect is 0.8

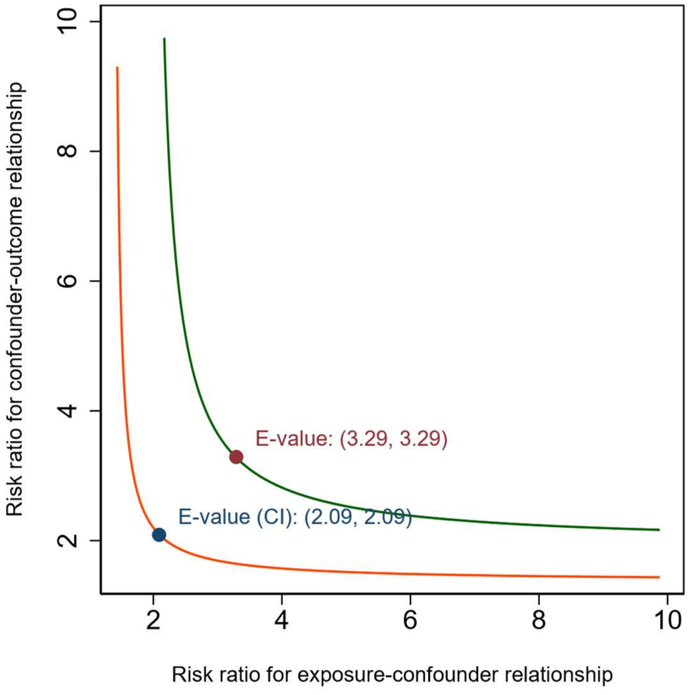

Propensity Score Sensitivity Analyses

The main analyses were re-run using propensity scores (PS) incorporated using inverse probability of treatment weights (IPTW). The PS were derived from a logistic (COPD) and multinomial logistic (asthma) model with the same covariates as the main cox regression models. Weights were created as 1/ps (ICS) and 1/(1-ps) (LABA/LAMA) in the COPD population (where ps is the probability of receiving ICS treatment) and 1/ps1 (SABA) 1/ps2 (Low/Medium dose ICS) and 1/ps3 (High-dose ICS) in the asthma population (where ps1 is the probability of receiving SABA only, ps2 the probability of receiving low/medium dose ICS and ps3 the probability of receiving high-dose ICS). These are Average Treatment Effect (ATE) weights, and were used in the PS-analyses presented in the main manuscript. Although we did not expect the Average Treatment Effect in the Treated (ATT) to be materially different from the ATE as we were not expecting that the effect of ICS on COVID-19 would vary strongly according to patient characteristics, we re-ran the analyses using ATT weights as well (results presented in supplementary materials only). These were created by multiplying the weights by the PS for the target treatment group; ICS (COPD) and High-dose ICS (asthma) respectively. Resulting weights are 1 (ICS) and ps/(1-ps) (LABA/LAMA) in the COPD population and ps3/ps1 (SABA), ps3/ps2 (Low-dose ICS) and 1 (High-Dose ICS) in the asthma population.

We then fit Cox regression models with robust standard errors in the weighted pseudopopulations. It should be noted that the 95%CI presented are conservative as they have been calculated assuming the weights are known and not estimated. We assessed covariate imbalance across treatment groups before and after weighting by calculating standardized differences using a modification of the stddiff program in Stata; code is provided in the github repo. We took a threshold of 0.1 on the absolute scale as a rough indication of potential for confounding by that covariate. We checked the distribution of weights; maximum weights were approximately 10 (copd) and 30 (asthma), and as we considered this unlikely to influence the outcomes given the size of the study populations we did not trim or truncate the weights. Overlap of the propensity score(s) across treatment groups was assessed graphically and by summarising by treatment group; no problems with lack of overlap were detected.

Table 13. Standardised Differences Before and After Weighting in the COPD population

Variable	Before Weighting	After Weighting
Male	0.01551821	0.00191297
Age (years)	-0.07235305	0.00269147
Evidence of obesity (4 categories)	0.01044024	0.00757069
Smoking status (missing set to non)	0.12745498	0.00364223
Index of Multiple Deprivation (IMD)	0.0182697	-0.00244183
Chronic kidney disease	0.01101934	0.00282707
Diagnosed hypertension	-0.02958052	0.00275994
Heart Failure	-0.0190057	-0.00318503
Other Heart Diseases	0.00710682	0.00409811
Diabetes Severity	-0.00559481	0.00904423
Cancer	0.00202028	0.00136973
Recent Statin	0.01933718	0.00526601
Flu vaccine	-0.02876889	0.00342335
Pneumococcal Vaccine	0.12430906	0.00098524
Exacerbation in last year	-0.15005884	0.00152897
Asthma ever	-0.37568988	-0.00070674
Immunosuppressed (combination algorithm)	-0.00005352	0.00254257

Table 14. Standardised Differences Before and After Weighting in the Asthma population

Variable	Before weighting		After weighting	
	ICS (Low/Medium Dose) vs SABA only	ICS (High Dose) vs SABA only	ICS (Low/Medium Dose) vs SABA only	ICS (High Dose) vs SABA only
Male	0.05439687	0.09800501	0.00197843	0.00276438
Age (years)	-0.27746089	-0.39707245	0.02452706	0.02578125
Evidence of obesity (4 categories)	-0.03539032	-0.19182719	0.01766467	0.01904679
Smoking status (missing set to non)	0.09949399	0.03244013	0.0040691	0.00752316
Index of Multiple Deprivation (IMD)	0.02705215	-0.07981947	0.01179471	0.01350516
Chronic kidney disease	-0.05007898	-0.09704192	0.02079124	0.0216233
Diagnosed hypertension	-0.11713768	-0.21525975	0.01228415	0.01407921
Heart Failure	-0.01761105	-0.07436071	0.00910582	0.01053839
Other Heart Diseases	-0.03398096	-0.10120148	0.01768929	0.01923399
Diabetes Severity	0.01362477	-0.08063203	0.01328383	0.01546454
Cancer	-0.03939077	-0.06008544	0.00442179	0.00492324
Recent Statin	-0.13082311	-0.22734816	0.01066121	0.0127148
Flu vaccine	-0.42027382	-0.50554283	0.02060794	0.01824828
Pneumococcal Vaccine	-0.08962935	-0.14003493	0.01801519	0.01724776
Exacerbation in last year	-0.15906407	-0.53182356	0.0074552	0.00917089
Immunosuppressed (combination algorithm)	0.01192759	0.00752758	0.00310104	0.00334448

Figure 17. COPD: Kernel Density Plot of PS by treatment, before weighting

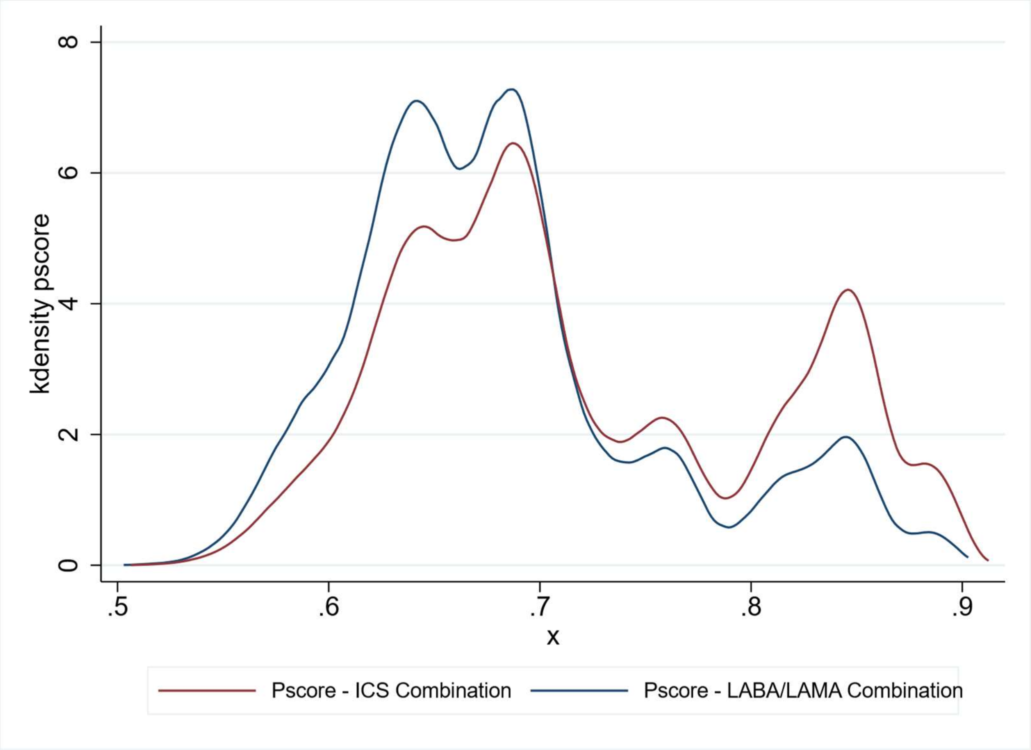

Figure 18. COPD: Kernel Density Plot of PS by treatment, after weighting (ATE)

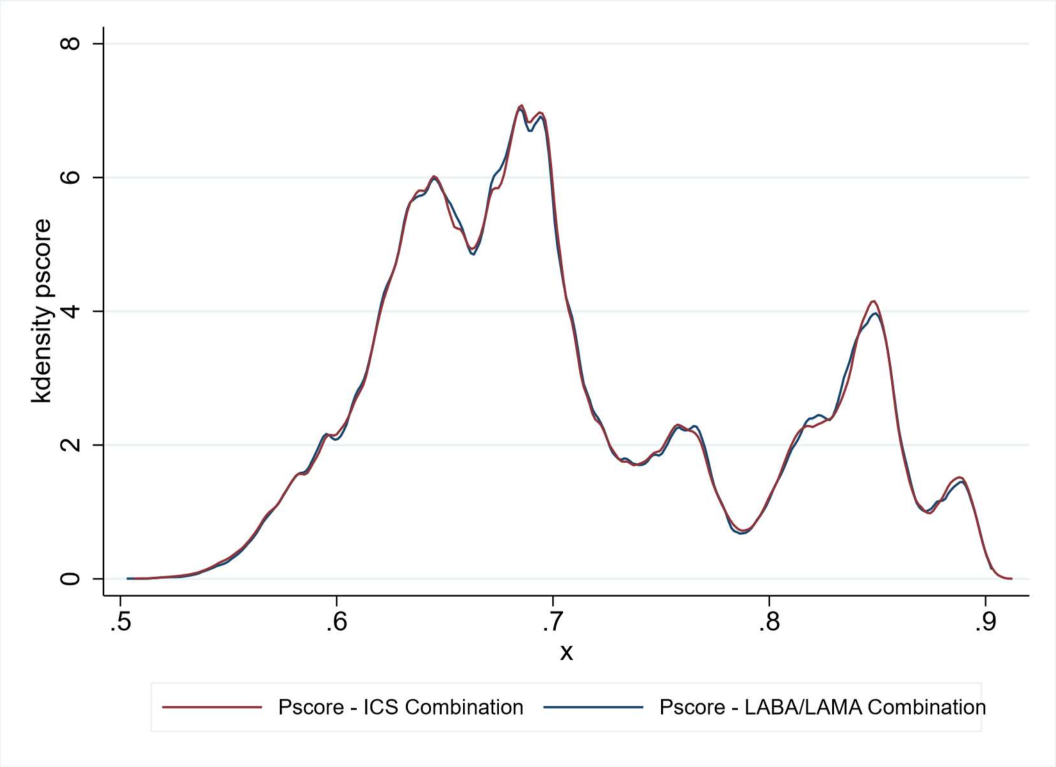

Figure 19. COPD: Kernel Density Plot of PS by treatment, after weighting (ATT)

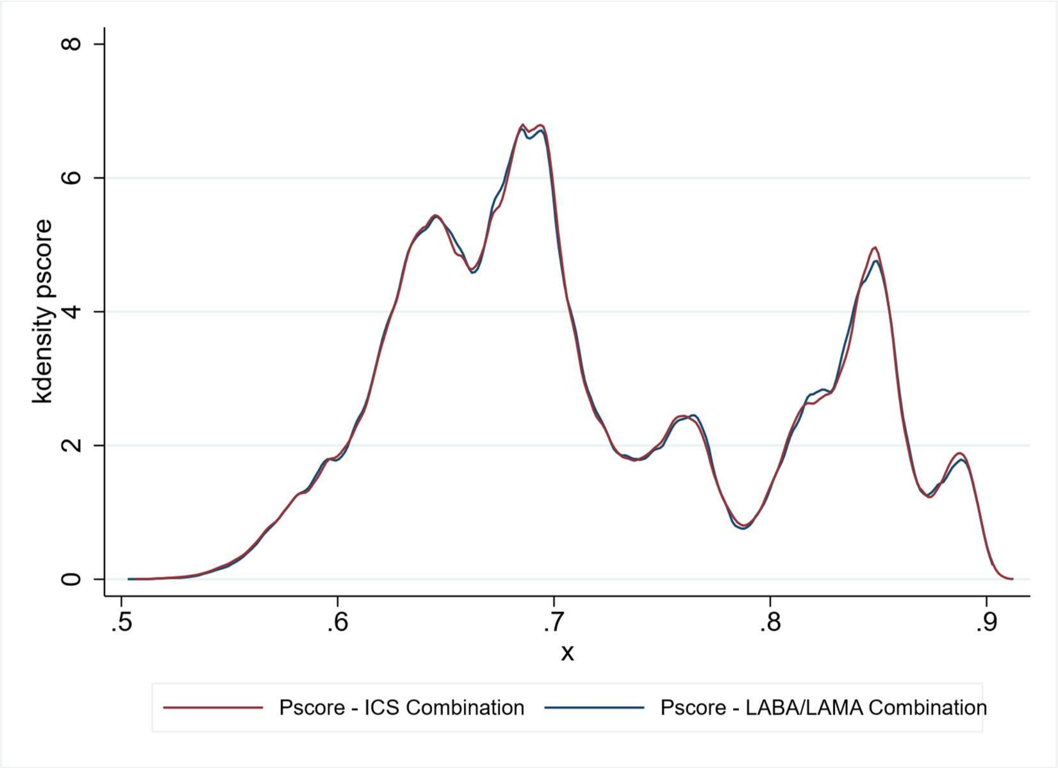

Figure 20. Asthma: Kernel Density Plot of Propensity Score for SABA only by treatment, before weighting

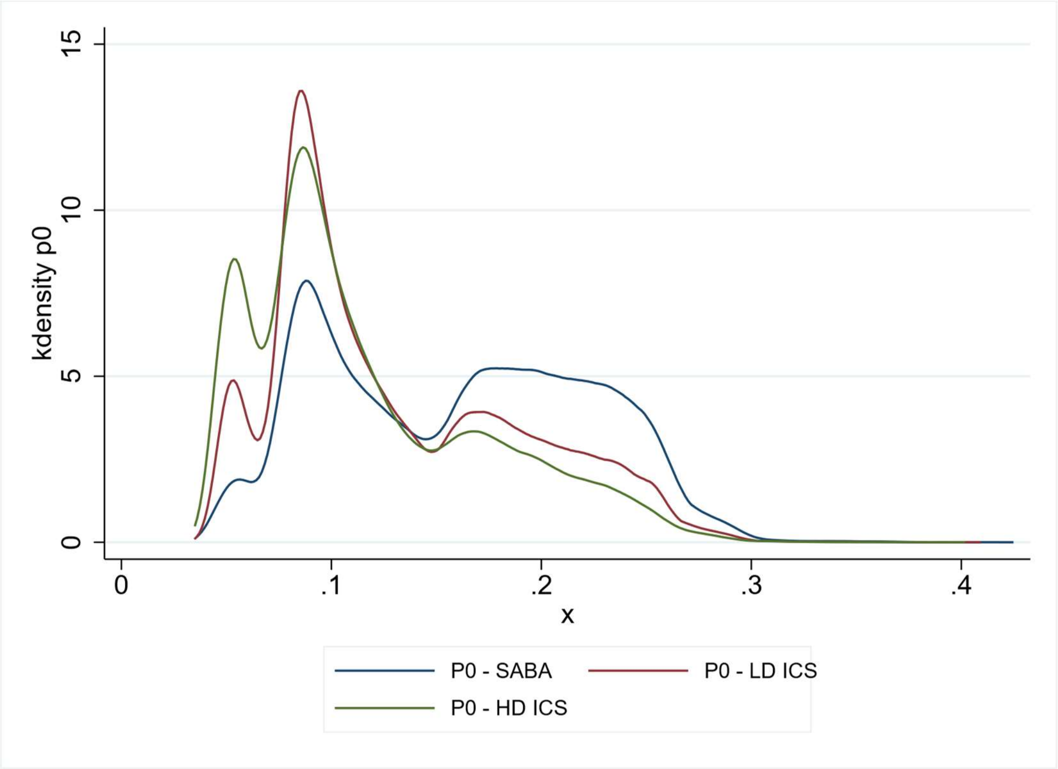

Figure 21. Asthma: Kernel Density Plot of Propensity Score for SABA only by treatment, after weighting (ATE)

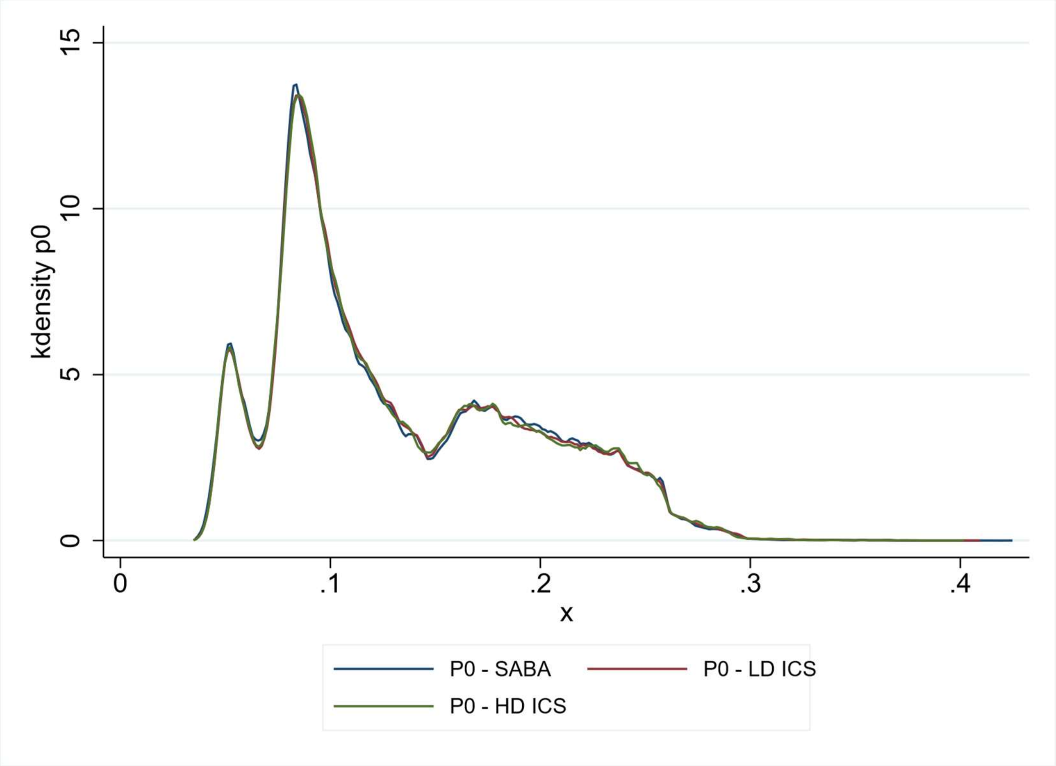

Figure 22. Asthma: Kernel Density Plot of Propensity Score for SABA only by treatment, after weighting (ATT)

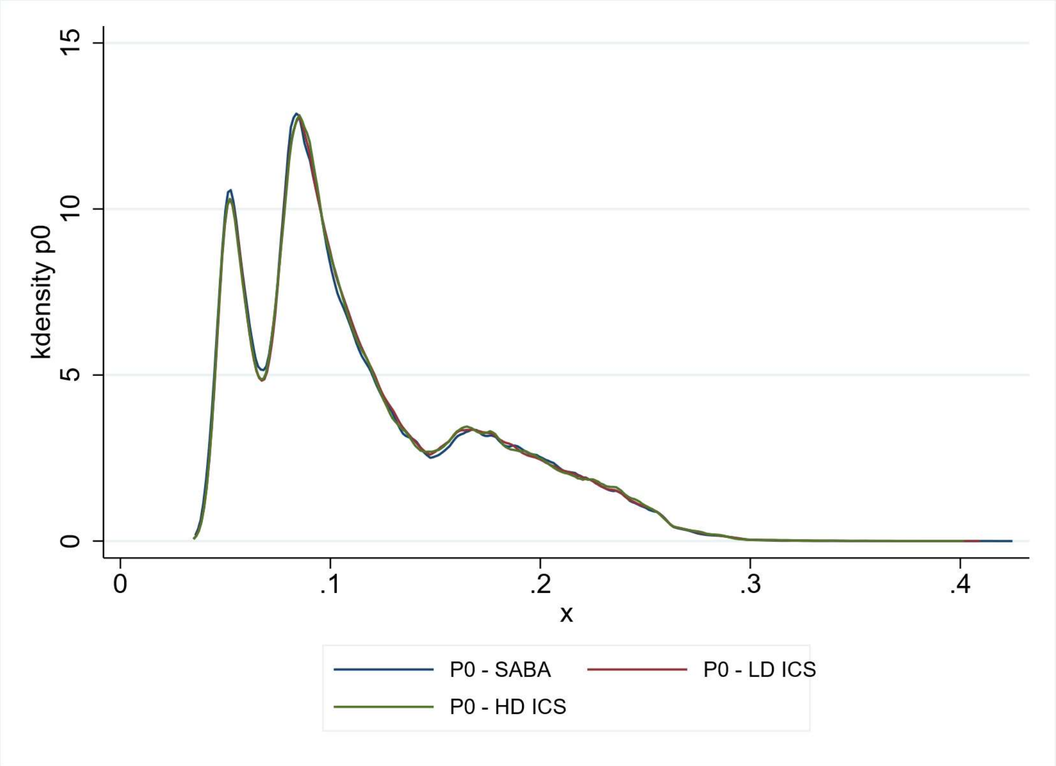

Figure 23. Asthma: Kernel Density Plot of Propensity Score for Low/Medium Dose ICS by treatment, before weighting

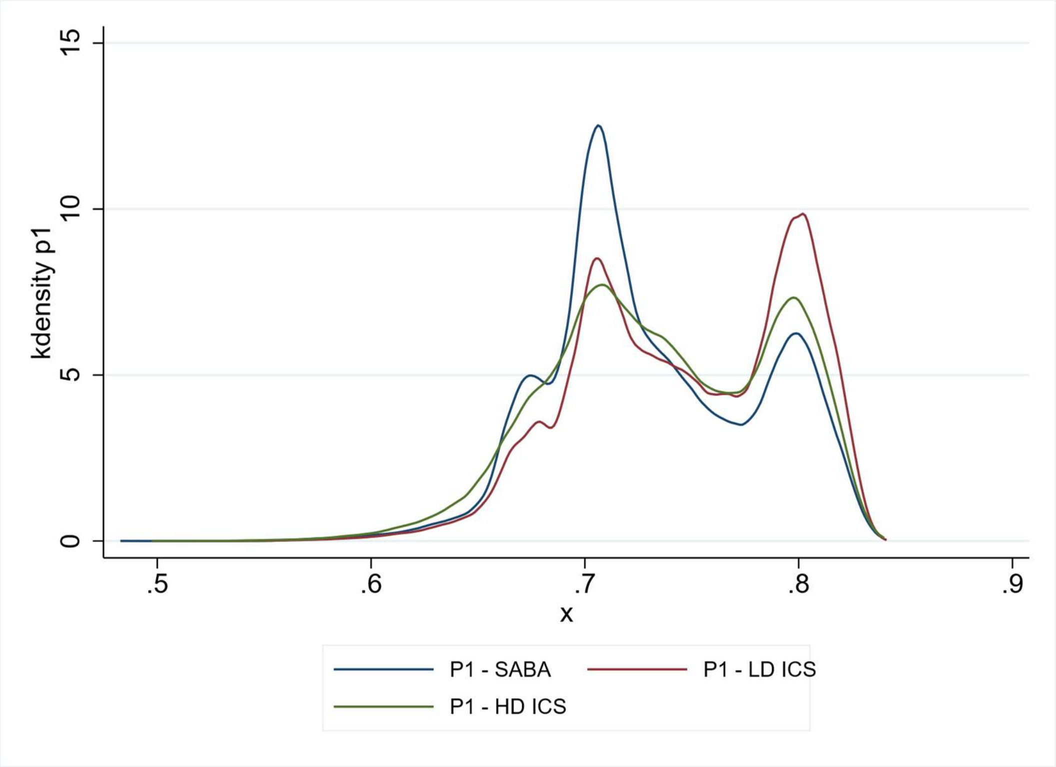

Figure 24. Asthma: Kernel Density Plot of Propensity Score for Low/Medium Dose ICS by treatment, after weighting (ATE)

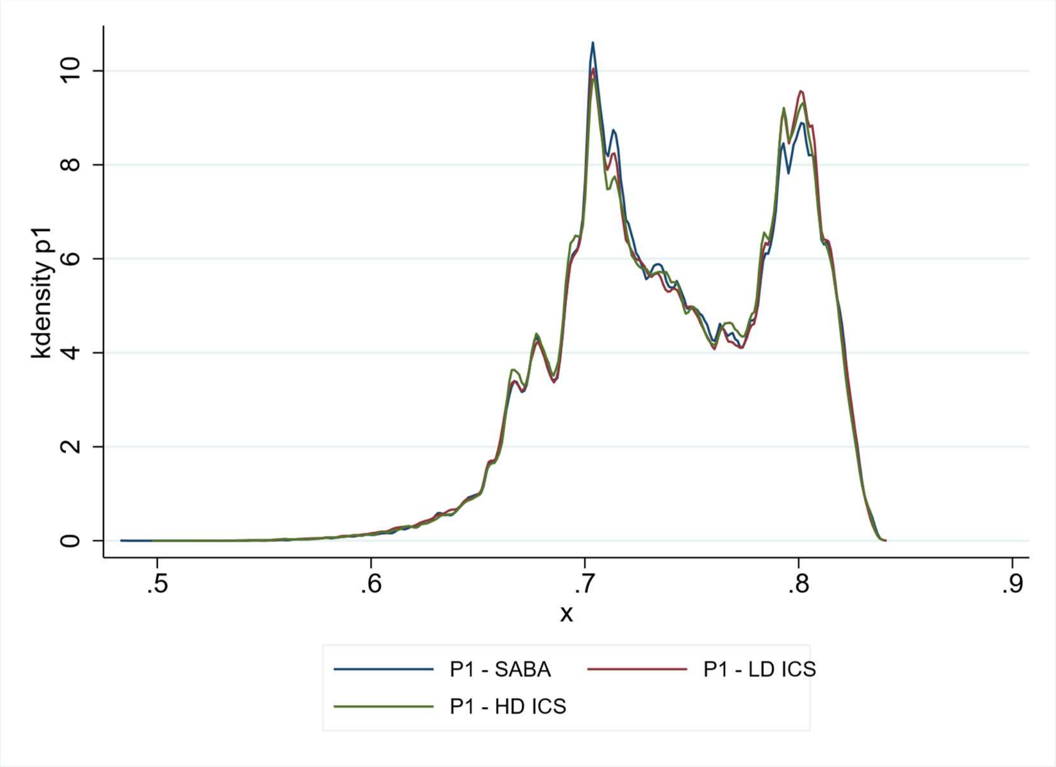

Figure 25.Asthma: Kernel Density Plot of Propensity Score for Low/Medium Dose ICS by treatment, after weighting (ATT)

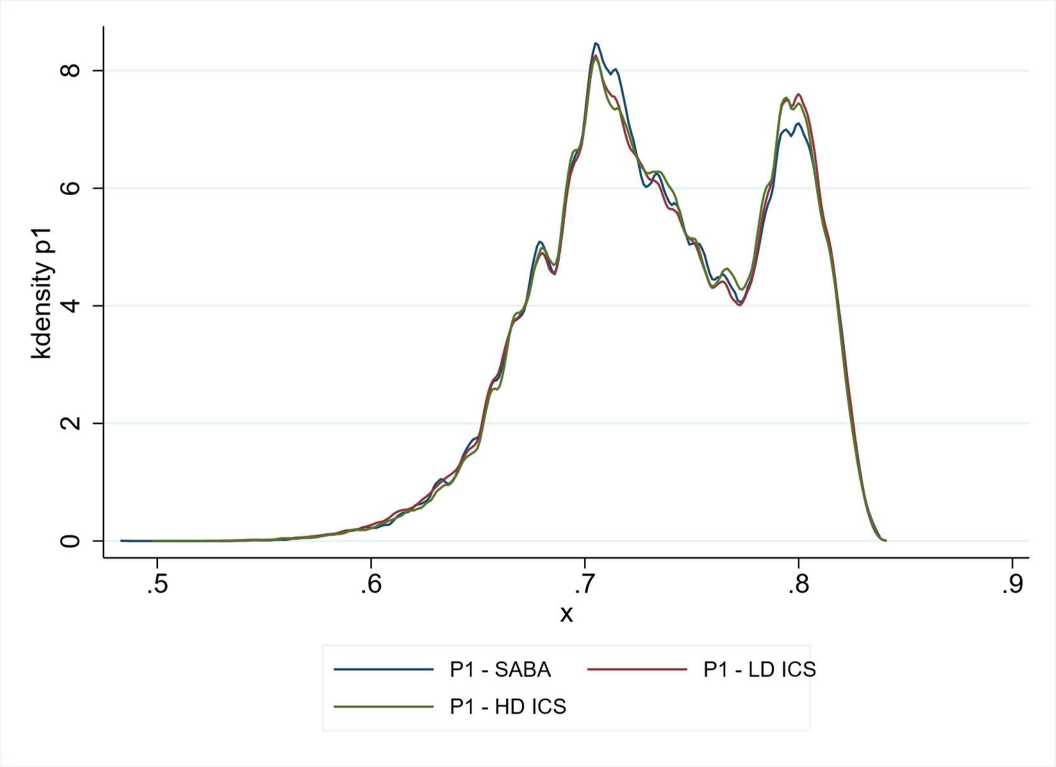

Figure 26. Asthma: Kernel Density Plot of Propensity Score for High Dose ICS by treatment, before weighting

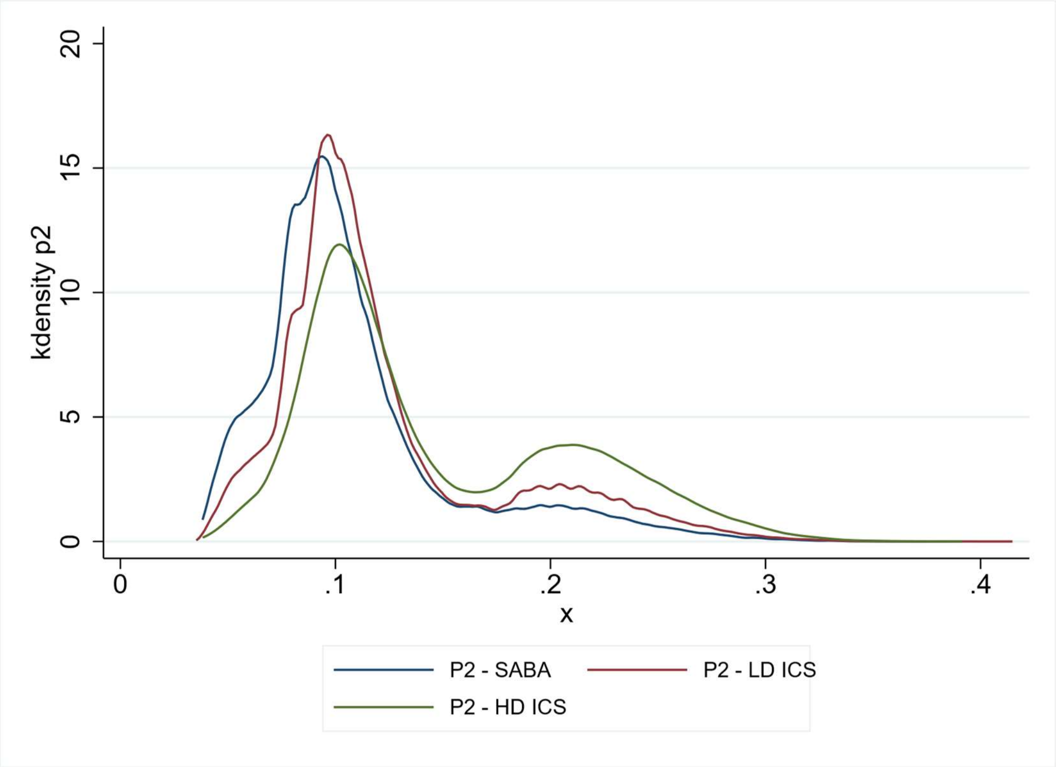

Figure 27. Asthma: Kernel Density Plot of Propensity Score for High Dose ICS by treatment, after weighting (ATE)

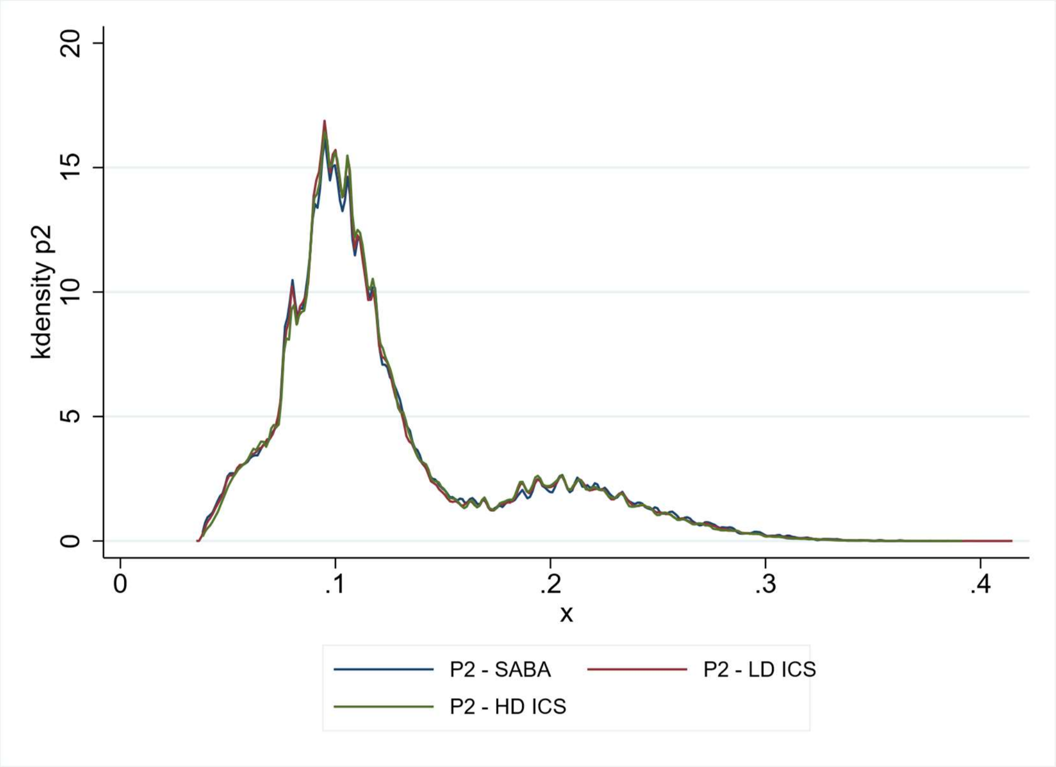

Figure 28. Asthma: Kernel Density Plot of Propensity Score for High Dose ICS by treatment, after weighting (ATT)

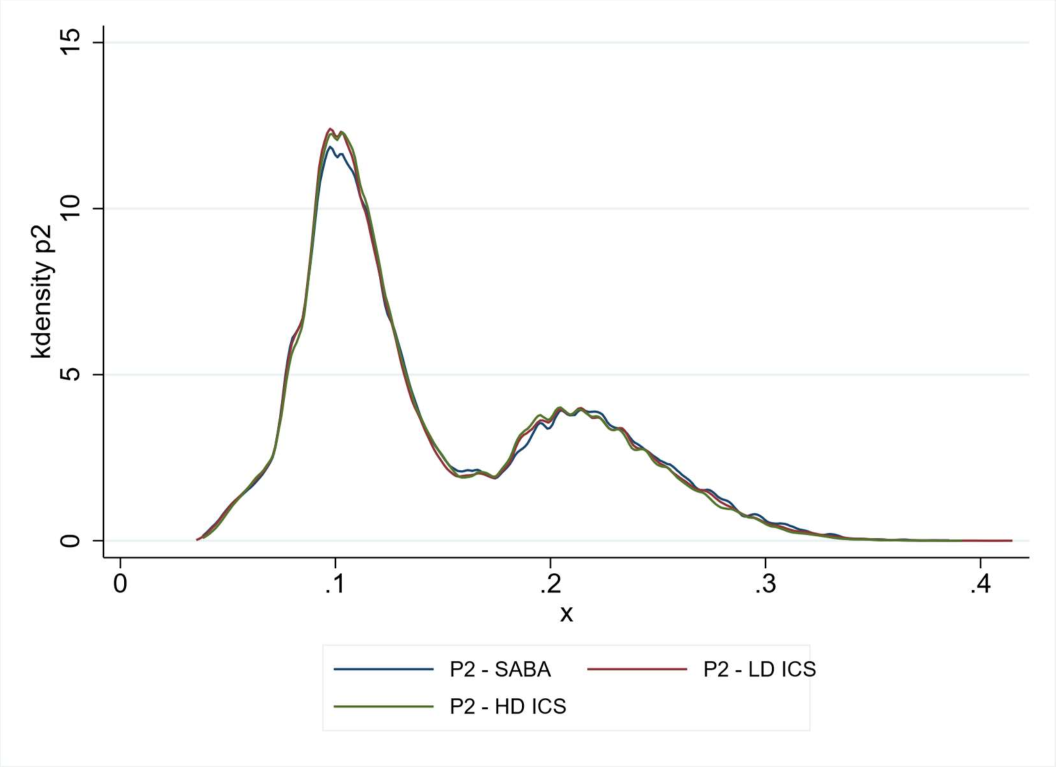

Table 15. Association between ICS and COVID-19 deaths in the COPD population - IPTW models

	ATE weights		ATT weights	
	HR	95% CI	HR	95% CI
LABA/LAMA Combination	1.00 (ref)		1.00 (ref)	
ICS Combination	1.44	1.13 - 1.84	1.45	1.13 - 1.86

Table 16. Association between ICS and COVID-19 deaths in the Asthma population - IPTW models

	ATE weights		ATT weights	
	HR	95% CI	HR	95% CI
SABA only	1.00 (ref)		1.00 (ref)	
ICS (Low/Medium Dose)	1.1	0.80 - 1.51	1.1	0.75 - 1.60
ICS (High Dose)	1.52	1.05 - 2.20	1.54	1.02 - 2.31

Table 17. Summary of available follow-up time, by treatment group in the COPD population

	Total	LABA/LAMA Combination	ICS Combination
Analysis time when record ends			
Median (IQR)	66 (66-66)	66 (66-66)	66 (66-66)
Mean (SD)	65.68 (3.59)	65.72 (3.39)	65.61 (3.95)
Min, Max	1, 66	1, 66	1, 66

Table 18. Summary of available follow-up time, by treatment group in the asthma population

	Total	SABA only	ICS (Low/Medium Dose)	ICS (High Dose)
Analysis time when record ends				
Median (IQR)	66 (66-66)	66 (66-66)	66 (66-66)	66 (66-66)
Mean (SD)	65.94 (1.51)	65.94 (1.64)	65.94 (1.59)	65.91 (1.95)
Min, Max	1, 66	1, 66	1, 66	1, 66

Table 19. STROBE and RECORD checklist

	Item No.	STROBE items	Location in manuscript where items are reported	RECORD items	Location in manuscript where items are reported
**Title and abstract**					
	1	(a) Indicate the study's design with a commonly used term in the title or the abstract (b) Provide in the abstract an informative and balanced summary of what was done and what was found	Title (study design)<br>Abstract (study design)	RECORD 1.1: The type of data used should be specified in the title or abstract. When possible, the name of the databases used should be included.<br><br>RECORD 1.2: If applicable, the geographic region and timeframe within which the study took place should be reported in the title or abstract.<br><br>RECORD 1.3: If linkage between databases was conducted for the study, this should be clearly stated in the title or abstract.	Abstract (type of data + names of databases)<br><br>Abstract (geographic region and timeframe)<br><br>Abstract (linkage specified)
**Introduction**					
Background rationale	2	Explain the scientific background and rationale for the investigation being reported	Introduction		Introduction
Objectives	3	State specific objectives, including any prespecified hypotheses	Introduction		Introduction
**Methods**					
Study Design	4	Present key elements of study design early in the paper	Methods		Methods
Setting	5	Describe the setting, locations, and relevant dates, including periods of recruitment, exposure, follow-up, and data collection	Methods		Methods
Participants	6	*(a) Cohort study* - Give the eligibility criteria, and the sources and methods of selection of participants. Describe methods of follow-up<br>*Case-control study* - Give the eligibility criteria, and the sources and methods of case ascertainment and control selection. Give the rationale for the choice of cases and controls<br>*Cross-sectional study* - Give the eligibility criteria, and the sources and methods of selection of participants<br><br>*(b) Cohort study* - For matched studies, give matching criteria and number of exposed and unexposed<br>*Case-control study* - For matched studies, give matching criteria and the number of controls per case	(a) Methods (Study Design, Study Population)<br>(b) NA	RECORD 6.1: The methods of study population selection (such as codes or algorithms used to identify subjects) should be listed in detail. If this is not possible, an explanation should be provided.<br><br>RECORD 6.2: Any validation studies of the codes or algorithms used to select the population should be referenced. If validation was conducted for this study and not published elsewhere, detailed methods and results should be provided.<br><br>RECORD 6.3: If the study involved linkage of databases, consider use of a flow diagram or other graphical display to demonstrate the data linkage process, including the number of individuals with linked data at each stage.	6.1. Codelists are publicly available.<br><br>6.2. Validation studies cited where relevant, deviations expalined (exacerbation identification only)<br><br>6.3. Not possible to evaluate, as linkage only conducted for individuals who died

Variables	7	Clearly define all outcomes, exposures, predictors, potential confounders, and effect modifiers. Give diagnostic criteria, if applicable.	Methods (Exposures, Outcomes, Covariates) Box 1 defines covariates, codelists and analytical code are publicly available.	RECORD 7.1: A complete list of codes and algorithms used to classify exposures, outcomes, confounders, and effect modifiers should be provided. If these cannot be reported, an explanation should be provided.	Box 1 defines covariates including algorithms where these were used, codelists and analytical code are publicly available and cited in the paper.
Data sources/ measurement	8	For each variable of interest, give sources of data and details of methods of assessment (measurement).<br>Describe comparability of assessment methods if there is more than one group	Box 1; analytical code (study_definition.py)		Box 1; analytical code (study_definition.py)
Bias	9	Describe any efforts to address potential sources of bias	Methods (sensitivity analyses)		Methods (sensitivity analyses)
Study size	10	Explain how the study size was arrived at	Methods (study population)		Methods (study population)
Quantitative variables	11	Explain how quantitative variables were handled in the analyses. If applicable, describe which groupings were chosen, and why	Methods (statistical methods)		Methods (statistical methods)
Statistical methods	12	(a) Describe all statistical methods, including those used to control for confounding<br>(b) Describe any methods used to examine subgroups and interactions<br>(c) Explain how missing data were addressed<br>(d) *Cohort study* - If applicable, explain how loss to follow-up was addressed<br>*Case-control study* - If applicable, explain how matching of cases and controls was addressed<br>*Cross-sectional study* - If applicable, describe analytical methods taking account of sampling strategy<br>(e) Describe any sensitivity analyses	a-c. Methods (covariates, statistical methods); Results.<br>d. N/A (study time frame so short LTFU was not considered an issue).<br>e. Methods (sensitivity analyses)		a-c. Methods (covariates, statistical methods); Results.<br>d. N/A (study time frame so short LTFU was not considered an issue).<br>e. Methods (sensitivity analyses)
Data access and cleaning methods		..	Methods, publicly available code, Data Sharing Statement	RECORD 12.1: Authors should describe the extent to which the investigators had access to the database population used to create the study population.<br><br>RECORD 12.2: Authors should provide information on the data cleaning methods used in the study.	Methods, publicly available code, Data Sharing Statement, Role of the Fuding source
Linkage		..		RECORD 12.3: State whether the study included person-level, institutional-level, or other data linkage across two or more databases. The methods of linkage and methods of linkage quality evaluation should be provided.	Methods
**Results**					
Participants	13	(a) Report the numbers of individuals at each stage of the study (*e.g.*, numbers potentially eligible, examined for eligibility, confirmed eligible, included in the study, completing follow-up, and analysed)	Supplemental materials, Figure 2-3.	RECORD 13.1: Describe in detail the selection of the persons included in the study (*i.e.,* study population selection) including filtering based on data quality, data availability and linkage. The selection of included	Supplemental materials, Figure 2-3.

		(b) Give reasons for non-participation at each stage.<br>(c) Consider use of a flow diagram		persons can be described in the text and/or by means of the study flow diagram.	
Descriptive data	14	(a) Give characteristics of study participants (*e.g.*, demographic, clinical, social) and information on exposures and potential confounders<br>(b) Indicate the number of participants with missing data for each variable of interest<br>(c) *Cohort study* - summarise follow-up time (*e.g.*, average and total amount)	a-c. Results, Table 1-2.		a-c. Results, Table 1-2.
Outcome data	15	*Cohort study* - Report numbers of outcome events or summary measures over time<br>*Case-control study* - Report numbers in each exposure category, or summary measures of exposure<br>*Cross-sectional study* - Report numbers of outcome events or summary measures	Results, Figure 1a-ab.		Results, Figure 1a-ab.
Main results	16	(a) Give unadjusted estimates and, if applicable, confounder-adjusted estimates and their precision (e.g., 95% confidence interval). Make clear which confounders were adjusted for and why they were included<br>(b) Report category boundaries when continuous variables were categorized<br>(c) If relevant, consider translating estimates of relative risk into absolute risk for a meaningful time period	a-c. Results		a-c. Results
Other analyses	17	Report other analyses done—e.g., analyses of subgroups and interactions, and sensitivity analyses	Results		Results
**Discussion**					
Key results	18	Summarise key results with reference to study objectives	Discussion		Discussion
Limitations	19	Discuss limitations of the study, taking into account sources of potential bias or imprecision. Discuss both direction and magnitude of any potential bias	Discussion	RECORD 19.1: Discuss the implications of using data that were not created or collected to answer the specific research question(s). Include discussion of misclassification bias, unmeasured confounding, missing data, and changing eligibility over time, as they pertain to the study being reported.	Discussion. Changing eligibility does not apply, as the study time period is so short (~2 months).
Interpretation	20	Give a cautious overall interpretation of results considering objectives, limitations, multiplicity of analyses, results from similar studies, and other relevant evidence	Discussion		Discussion
Generalisability	21	Discuss the generalisability (external validity) of the study results	Discussion		Discussion
**Other Information**					

Funding	22	Give the source of funding and the role of the funders for the present study and, if applicable, for the original study on which the present article is based	Administrative (Funding)		Administrative (Funding)
Accessibility of protocol, raw data, and programming code		..	Methods (software and reproducibility)	RECORD 22.1: Authors should provide information on how to access any supplemental information such as the study protocol, raw data, or programming code.	Methods (software and reproducibility)

*Reference: Benchimol EI, Smeeth L, Guttmann A, Harron K, Moher D, Petersen I, Sørensen HT, von Elm E, Langan SM, the RECORD Working Committee. The REporting of studies Conducted using Observational Routinely-collected health Data (RECORD) Statement. *PLoS Medicine* 2015; in press.

*Checklist is protected under Creative Commons Attribution (CC BY) license.

Information Governance

NHS England is the data controller; TPP is the data processor; and the key researchers on OpenSAFELY are acting on behalf of NHS England. This implementation of OpenSAFELY is hosted within the TPP environment which is accredited to the ISO 27001 information security standard and is NHS IG Toolkit compliant;[48,49] patient data has been pseudonymised for analysis and linkage using industry standard cryptographic hashing techniques; all pseudonymised datasets transmitted for linkage onto OpenSAFELY are encrypted; access to the platform is via a virtual private network (VPN) connection, restricted to a small group of researchers; the researchers hold contracts with NHS England and only access the platform to initiate database queries and statistical models; all database activity is logged; only aggregate statistical outputs leave the platform environment following best practice for anonymisation of results such as statistical disclosure control for low cell counts.[50] The OpenSAFELY research platform adheres to the data protection principles of the UK Data Protection Act 2018 and the EU General Data Protection Regulation (GDPR) 2016. In March 2020, the Secretary of State for Health and Social Care used powers under the UK Health Service (Control of Patient Information) Regulations 2002 (COPI) to require organisations to process confidential patient information for the purposes of protecting public health, providing healthcare services to the public and monitoring and managing the COVID-19 outbreak and incidents of exposure.[51] Taken together, these provide the legal bases to link patient datasets on the OpenSAFELY platform. GP practices, from which the primary care data are obtained, are required to share relevant health information to support the public health response to the pandemic, and have been informed of the OpenSAFELY analytics platform.
